# Implantation of engineered adipocytes suppresses tumor progression in cancer models

**DOI:** 10.1038/s41587-024-02551-2

**Published:** 2025-02-04

**Authors:** Hai P. Nguyen, Kelly An, Yusuke Ito, Bhushan N. Kharbikar, Rory Sheng, Breanna Paredes, Elizabeth Murray, Kimberly Pham, Michael Bruck, Xujia Zhou, Cassandra Biellak, Aki Ushiki, Mai Nobuhara, Sarah L. Fong, Daniel A. Bernards, Filipa Lynce, Deborah A. Dillon, Mark Jesus M. Magbanua, Laura A. Huppert, Heinz Hammerlindl, Jace Anton Klein, Luis Valdiviez, Oliver Fiehn, Laura Esserman, Tejal A. Desai, Sook Wah Yee, Jennifer M. Rosenbluth, Nadav Ahituv

**Affiliations:** 1 Department of Bioengineering and Therapeutic Sciences, University of California San Francisco, San Francisco, CA USA; 2 Institute for Human Genetics, University of California San Francisco, San Francisco, CA USA; 3 Department of Nutritional Sciences, University of Texas at Austin, Austin, TX USA; 4 Division of Hematology/Oncology, Department of Medicine, University of California San Francisco, San Francisco, CA USA; 5 Department of Medical Oncology, Dana-Farber Cancer Institute, Boston, MA USA; 6 Harvard Medical School, Boston, MA USA; 7 Department of Pathology, Brigham and Women’s Hospital, Boston, MA USA; 8 Department of Laboratory Medicine, University of California San Francisco, San Francisco, CA USA; 9 Department of Pharmaceutical Chemistry, University of California San Francisco, San Francisco, CA USA; 10 University of California Davis West Coast Metabolomics Center, Davis, CA USA; 11 Department of Surgery, University of California San Francisco, San Francisco, CA USA; 12 School of Engineering, Brown University, Providence, RI USA; 13 Chan Zuckerberg Biohub, San Francisco, CA USA

**Keywords:** Drug development, Cancer genetics

## Abstract

Tumors exhibit an increased ability to obtain and metabolize nutrients. Here, we implant engineered adipocytes that outcompete tumors for nutrients and show that they can substantially reduce cancer progression, a technology termed adipose manipulation transplantation (AMT). Adipocytes engineered to use increased amounts of glucose and fatty acids by upregulating *UCP1* were placed alongside cancer cells or xenografts, leading to significant cancer suppression. Transplanting modulated adipose organoids in pancreatic or breast cancer genetic mouse models suppressed their growth and decreased angiogenesis and hypoxia. Co-culturing patient-derived engineered adipocytes with tumor organoids from dissected human breast cancers significantly suppressed cancer progression and proliferation. In addition, cancer growth was impaired by inducing engineered adipose organoids to outcompete tumors using tetracycline or placing them in an integrated cell-scaffold delivery platform and implanting them next to the tumor. Finally, we show that upregulating *UPP1* in adipose organoids can outcompete a uridine-dependent pancreatic ductal adenocarcinoma for uridine and suppress its growth, demonstrating the potential customization of AMT.

## Abstract (web summary)

Adipose manipulation transplantation can reduce tumor growth and proliferation in vitro and in mouse models.

## Main

Tumors are complex tissues composed of cancerous and non-cancerous cells in a hypoxic and nutrient-deprived microenvironment. The tumor microenvironment contains heterogeneous cell populations, including immune cells, mesenchymal support cells and matrix components that contribute to tumor growth and progression^1^. To survive this environment, tumors are capable of reprogramming metabolic pathways to better use available substrates in the surrounding tumor microenvironment, ultimately becoming dependent on these pathways for continued growth and survival^2^. In contrast to normal cells, the main pathway of glucose metabolism in cancer cells is aerobic glycolysis, termed the Warburg effect^3^. Glucose uptake and lactate production are increased in these cells, even in the presence of oxygen and functional mitochondria^3^. The increase in glycolytic flux allows glycolytic intermediates to supply subsidiary pathways to fulfil the metabolic demands of proliferating cells. During hypoxia, cancer cells also undergo metabolic reprogramming to increase lipid use, as fatty acids produce twice the energy of glucose^4,5^.

There have been many efforts to target cancer glucose and fatty acid metabolism for therapeutic purposes. For glycolysis, these include drugs that target hexokinase 2 (HK2), which is involved in the initial steps in glycolysis, using ATP from the mitochondria to phosphorylate glucose, or drugs that target glucose transporters (GLUT1 and GLUT4)^2,6–8^. Several drugs are also used to target lipid metabolism in cancer^9–12^. These include drugs that target lipid uptake (targeting proteins such as LXR, CD36 and FABP4/5), lipogenic enzymes (such as ACC, ACLY, FASN and SCD1) and proteins involved in intracellular lipid homeostasis (such as CPT1A, PPARG and others)^13,14^. In addition, recent work has shown that cold activation of brown adipose tissue (BAT), which dissipates energy by non-shivering thermogenesis, increases adipocyte glucose uptake and lipid metabolism and significantly inhibits tumor growth^15^. However, situating cancer patients in cold conditions for extended periods is challenging.

Here, we set out to develop a therapeutic approach, termed adipose manipulation transplantation (AMT), that uses two unique abilities of white adipose tissue (WAT). First, it can be readily extracted in the clinic by liposuction and implanted through reconstructive surgery; second, it can change into a BAT-like tissue, called browning or beiging^16,17^, by upregulating essential transcriptional regulators or enzymes, such as the uncoupling protein 1 (*UCP1*)*, PPARG* coactivator 1 alpha (*PPARGC1A*) or PR/SET domain 16 (*PRDM16*) genes^18–33^. Similar to BAT, beige adipocytes have the capacity to convert energy to heat and contribute to whole-body energy expenditure^34^. We show that CRISPR activation (CRISPRa) of either *UCP1*, *PRDM16* or *PPARGC1A* induces browning, subsequently increasing glucose and fat metabolism in human white adipocytes and adipose organoids. Co-culturing of these CRISPRa-modulated adipocytes with various cancer cell lines (breast, colon, pancreatic or prostate cancer) significantly suppresses cancer cell proliferation as well as decreases glucose uptake, glycolysis and fatty acid oxidation (FAO) capacity in the cancer cells. Subcutaneously co-transplanting CRISPRa-modulated human adipose organoids and cancer cell xenografts (two different breast cancer lines, pancreatic or prostate) into immune-compromised mice leads to significantly reduced tumor size with decreased hypoxia and angiogenesis. Implantation of engineered adipose organoids into pancreatic or breast cancer genetic mouse models significantly suppresses cancer progression. Furthermore, in the breast cancer model, we show that their implantation both near and distal to the tumor leads to similar results. To further demonstrate the therapeutic potential of this approach, we show that adipocytes isolated from resected human breast tissues can be similarly manipulated with CRISPRa and can inhibit the growth of patient-derived breast cancer organoids as well as the proliferation of high-risk non-cancerous breast tissues such as those from patients with *BRCA1/2* mutations. In addition, we also show how induction of CRISPRa using tetracycline or the implantation of modulated adipose organoids in an integrated cell-scaffold delivery platform can control this therapeutic approach in a reversible manner. Finally, to show that this approach can be customizable for other tumor-associated metabolic programs, we show how CRISPRa upregulation of uridine phosphorylase 1 (*UPP1*) in adipose organoids can suppress xenograft growth of a uridine-dependent pancreatic ductal adenocarcinoma (PDA)^35^. Combined, our results introduce a cancer therapeutic approach that has the potential to treat numerous cancer types.

## Results

### CRISPRa browning of human white adipocytes

**Extended Data Fig. 1:**
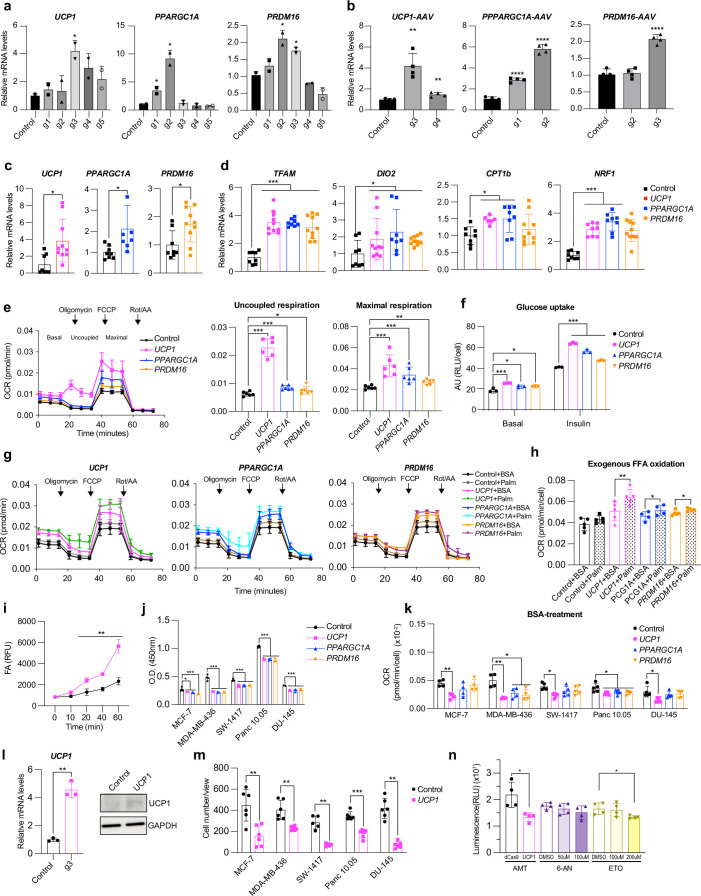
CRISPRa upregulation of *UCP1*, *PPARGC1A*, and *PRDM16* in human adipocytes suppresses cancer progression in various cancer types. **a**, qRT-PCR of *UCP1*, *PPARGC1A*, and *PRDM16* in white adipocytes transfected with CRISPRa using five different gRNAs per gene (n = 2 biological replicates). **b**, qRT-PCR of *UCP1*, *PPARGC1A*, and *PRDM16* in human adipocytes transduced by AAV9-CRISPRa with the top two gRNAs per gene (n = 4 biological replicates). **c**, qRT-PCR of *UCP1*, *PPARGC1A*, and *PRDM16* in human white adipocytes transduced with CRISPRa targeting *UCP1*, *PPARGC1A*, and *PRDM16* (n = 8–10 biological replicates). **d**, qRT-PCR of *TFAM*, *DIO2*, *CPT1b*, and *NRF1* in CRISPRa-modulated adipocytes (n = 8–10 biological replicates). **e**, Oxygen consumption rate (OCR) of CRISPRa-modulated adipocytes measured by the seahorse assay (n = 6 biological replicates). Uncoupled OCR was measured under oligomycin treatment, while maximal OCR was measured under FCCP. **f**, Glucose uptake of CRISPRa-modulated adipocytes with or without insulin (n = 3 biological replicates). **g**, OCR of CRISPRa-modulated cells measured by the seahorse assay in BSA- or BSA-Palmitate- medium (n = 5 biological replicates). **h**, Exogenous fatty acid oxidation of CRISPRa-modulated adipocytes calculated by the difference of area under the curve of OCR between BSA- and BSA-Palmitate media upon FCCP treatment (n = 4-5 biological replicates). **i**, Fatty acid (FA) uptake of control and *UCP1*-CRISPRa-modulated adipocytes (n = 4 biological replicates). **j**, BrdU incorporation of five cancers cell lines co-cultured with CRISPRa-modulated adipocytes (n = 4-5 biological replicates). **k**, OCR under FCCP of cancer cells co-cultured with CRISPRa-modulated cells measured by the seahorse assay in BSA- medium (n = 4-5 biological replicates). **l**, qRT-PCR (n= 3 biological replicates) and immunoblotting (n = 2 biological replicates; uncropped images in Source Data Extended Data Fig. 1) for *UCP1* in primary adipocytes transduced with *UCP1-*CRISPRa. **m**, Cell numbers of various cancer cells co-cultured with CRISPRa-modulated primary adipocytes (n = 5-6 biological replicates). **n**, Cell viability assay on MCF-7 cells treated with CRISPRa-*UCP1*-AAV adipocytes (AMT), 6-aminonicotinamide (6-AN; purple bars) at 50 μM, 100 μM and DMSO as negative control and Etomoxir (ETO; yellow bars) at100 μM, 200 μM and DMSO as negative control (n = 4 biological replicates). All statistical tests in d,e,f,j,k were carried out using a one-way ANOVA and the rest using a two tailed t-test. Data are represented as mean ± standard deviation (SD) *≤0.05, **≤0.01, ***≤0.001. Source data

To induce browning in human adipocytes, we used CRISPRa to upregulate *UCP1*, *PPARGC1A* or *PRDM16*, all known genes involved in BAT development and function. Using CRISPick^36^, we designed five guide RNAs (gRNAs) targeting each gene’s promoter and cloned them into an adeno-associated virus (AAV)-based expression vector. Differentiated adipocytes derived from human white preadipocytes were co-transfected with the gRNAs along with a *Staphylococcus aureus* endonuclease-deficient Cas9 (dCas9) fused to the VP64 transcriptional activator. We used an *S.* *aureus* dCas9 due to its smaller size and the VP64 transcriptional activator (which carries four copies of VP16, a herpes simplex virus type 1 transcriptional activator^37^) because it provides moderate gene upregulation and is small enough to fit into AAV, which has a 4.7 kb optimal packaging capacity^38^. To generate mature human adipocytes, preadipocytes were subjected to adipocyte differentiation using a cocktail of 3-isobutyl-1-methylxanthine (IBMX), dexamethasone and insulin before being subjected to CRISPRa. After 4 days, we measured gene expression by quantitative PCR with reverse transcription (RT–qPCR), finding several gRNAs to significantly increase the expression levels of *UCP1*, *PPARGC1A* and *PRDM16* in the transfected cells compared to the control cells (treated with dCas9–VP64 only) (Extended Data Fig. 1a). We selected the top two gRNAs for each gene for AAV serotype 9 (AAV9) packaging. We used AAV9 because it was shown to effectively infect various adipose depots^39^. We infected human differentiated adipocytes with these viruses along with AAV9 dCas9–VP64, finding for each gene at least one gRNA that significantly increased expression levels compared to the dCas9–VP64-only control (Extended Data Fig. 1b). We used the top-activating gRNA AAV for all subsequent experiments.

We next examined whether our CRISPRa treatment increases browning in these human white adipocytes. Human adipocytes transduced with the top gRNA for *UCP1*, *PRDM16* or *PPARGC1A* showed significantly increased expression of their target genes compared to dCas9–VP64-only infected cells (Extended Data Fig. 1c). In addition, we also observed increased mRNA levels for brown fat marker genes, including *TFAM*, *DIO2*, *CPT1b* and *NRF1* upon upregulation of either of the three genes (Extended Data Fig. 1d); *PRDM16* CRISPRa did not show upregulation of *CPT1b* and *NRF1*. We next examined the oxygen consumption rate (OCR) in these cells using Seahorse (Methods), by initially blocking oxygen consumption and then adding 1 μM oligomycin, followed by the introduction of 1 μM carbonyl cyanide-p-trifluoromethoxy-phenylhydrazone (FCCP) to measure the maximal respiratory capacity. We found that CRISPRa-AAV treatment targeting all these genes increased overall OCR levels in human white adipocytes, with *UCP1*-gRNA-AAV-treated cells having the largest increase (Extended Data Fig. 1e). In addition, these cells showed increased uncoupled respiration (under oligomycin, an ATP synthase inhibitor), indicating a brown fat-like phenotype (Extended Data Fig. 1e). CRISPRa-treated cells also had elevated maximal respiration following FCCP treatment (Extended Data Fig. 1e). Furthermore, these CRISPRa-AAV-engineering adipocytes showed increased glucose uptake in both basal and insulin-stimulated conditions (Extended Data Fig. 1f). We also tested the FAO capacity of these cells by performing a similar Seahorse OCR assay. In both BSA and BSA-conjugated-palmitate (saturated fatty acid complex) media, we found that our CRISPRa-treated adipocytes had an overall OCR increase in BSA-conjugated-palmitate (Extended Data Fig. 1g). Under FCCP treatment, in which the increase of OCR in palmitate-containing media compared to BSA-containing media is thought to be caused by exogenous FAO, we found that upregulation of either gene increased exogenous FAO capacity in human adipocytes (Extended Data Fig. 1h). Similarly, *UCP1*-upregulated human adipocytes had the largest increase in FAO. We also found that *UCP1*-upregulated human adipocytes had higher fatty acid uptake than the dCas9–VP64 control cells (Extended Data Fig. 1i). Taken together, we show that AAV-based CRISPRa upregulation of *UCP1*, *PPARGC1A* or *PRDM16* induces browning in human adipocytes, leading them to have increased glucose uptake and FAO.

### CRISPRa-modulated adipocytes suppress tumor growth in vitro

**Fig. 1:**
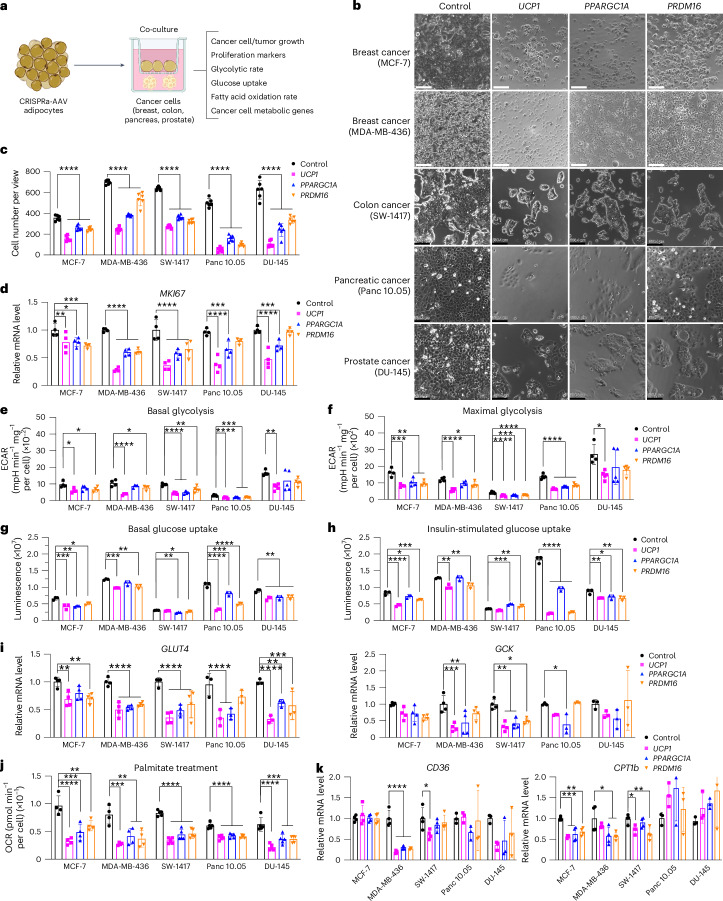
CRISPRa-modulated adipocytes inhibit cancer cell growth in vitro. **a**, Schematic of the co-culturing model of cancer cells and CRISPRa-treated adipocytes using Transwell plates and their subsequent phenotyping (created with BioRender.com). **b**, Representative images of cancer cells, including breast (MCF-7, MDA-MB-436), colon (SW-1417), pancreatic (Panc 10.05) and prostate cancer (DU-145), that were co-cultured with CRISPRa-upregulating *UCP1*, *PPARGC1a* and *PRDM16* or control (dCas9–VP64 only) adipocytes. Scale bars, 530.4 μm. **c**, Cancer cell number per view of image (four images or replicates per condition). **d**, RT–qPCR of the proliferation marker gene *MKI67* for cancer cells co-cultured with CRISPRa-modulated adipocytes (*n* = 4 biological replicates). **e**, Basal glycolysis measured by calculating the area under the curve of ECAR upon glucose treatment (*n* = 4–5 biological replicates). **f**, Maximal glycolysis measured by calculating the area under the curve of ECAR upon oligomycin treatment (*n* = 4–5 biological replicates). **g**,**h**, Glucose uptake of cancer cells co-cultured with CRISPRa-modulated adipocytes without (**g**) or with (**h**) insulin (*n* = 3 biological replicates). **i**, RT–qPCR of glucose transporter *GLUT4* and glycolytic enzyme *GCK* in cancer cells (*n* = 3–4 biological replicates). **j**, Exogenous FAO of cancer cells calculated by the difference of area under the curve of OCR of BSA-palmitate media upon FCCP treatment (*n* = 4 biological replicates). **k**, RT–qPCR of fatty acid transporter *CD36* and fatty acid regulatory transporter *CPT1b* in cancer cells that were co-cultured with CRISPRa-treated adipocytes (*n* = 3–4 biological replicates). All statistical tests were carried out using a one-way ANOVA and data are represented as mean ± s.d. *P ≤ 0.05; **P ≤ 0.01; ***P ≤ 0.001; ****P ≤ 0.0001.

We next evaluated whether our CRISPRa ‘browned’ adipocytes could inhibit cancer growth in vitro using a co-culturing system (Fig. 1a). We initially treated differentiated adipocytes with CRISPRa-AAV for either of the three genes (*UCP1*, *PPARGC1A* or *PRDM16*) and replated these adipocytes on the top chamber of a 12-well or 24-well Transwell plate, which has inserts of 0.4 µm membranes, so that the adipocytes did not contact the cells on the lower chamber (Fig. 1a). In the lower chamber, we grew five different cancer cell lines: breast cancer cells MCF-7 (estrogen receptor-positive (ER^+^), progesterone receptor-positive (PR^+^), glucocorticoid receptor-positive (GR^+^)) and MDA-MB-436 (triple negative), colon cancer (SW-1417), pancreatic cancer (Panc 10.05) and prostate cancer (DU-145). As a negative control, we used adipocytes infected with dCas9–VP64 only. After 3 days, we observed that all five cancer cell lines that were co-cultured with *UCP1*, *PPARGC1A* or *PRDM16* CRISPRa-treated human adipocytes showed significantly lower cell numbers than cancer cells co-cultured with dCas9–VP64-treated adipocytes (Fig. 1b). We also found that the number of cancer cells co-cultured with CRISPRa-treated adipocytes was threefold to fivefold lower than cancer cells co-cultured with the control adipocytes (Fig. 1c). By RT–qPCR, we observed that all cancer cells co-cultured with CRISPRa-treated adipocytes had significantly reduced levels of the proliferation marker *MKI67* (other than Panc 10.05 and DU-145 treated with *PRDM16* CRISPRa) compared to control cells, with *UCP1*-CRISPRa having the greatest effect (Fig. 1d). We also performed a BrdU incorporation assay, finding that after 24 h, CRISPRa co-cultured cancer cells showed significantly reduced proliferation compared to cells co-cultured with control adipocytes (Extended Data Fig. 1j).

We next analyzed the glucose and fatty acid metabolism of the co-cultured cancer cells. To measure glycolysis, we used the extracellular acidification rate (ECAR) assay in which the basal glycolytic rate is measured by adding glucose and the maximal glycolytic rate is measured by oligomycin A addition. We found that most cancer cell lines co-cultured with CRISPRa-AAV-treated adipocytes showed a significant reduction in both basal and maximal glycolytic rate (Fig. 1e,f) and lower glucose uptake in both basal and insulin conditions (Fig. 1g,h). Using RT–qPCR, we also found that the expression of key glycolysis genes, such as *GCK* and *GLUT4*, a major glucose transporter, was significantly lower in most cancer cells co-cultured with CRISPRa-AAV adipocytes than in the negative control (Fig. 1i). We also examined FAO using Seahorse. In BSA-only media, we found that all cancer cell lines that were co-cultured with *UCP1*-CRISPRa-modulated adipocytes had lower OCR. For *PPARGC1A* and *PRDM16* CRISPRa-modulated adipocytes, this was only observed in MDA-MB-436 and Panc 10.05 cells (Extended Data Fig. 1k). In palmitate-containing media, we found that all five cancer cell lines that were co-cultured with CRISPRa-AAV adipocytes had reduced FAO compared to the negative control (Fig. 1j). Moreover, using RT–qPCR, we found in several CRISPRa conditions that the cancer cells had lower expression of both *CD36*, a fatty acid transporter on the cell membrane, and *CPT1b*, a key regulator of FAO in the mitochondria, further confirming decreased FAO (Fig. 1k). To show that this approach works with other adipocyte lines, we also upregulated *UCP1* in human adipocytes differentiated from primary preadipocytes (Extended Data Fig. 1l) and found that they inhibit tumor growth of all five cancer cells (Extended Data Fig. 1m). Combined, our data shows that our CRISPRa-modulated adipocytes reduce glycolysis and FAO in five different cancer cell lines and can significantly suppress cancer growth.

We next compared the tumor suppression capability of CRISPRa-AAV-modulated adipocytes to known metabolic cancer drugs. We grew MCF-7 cells and treated them with the following: (1) CRISPRa-*UCP1*-AAV adipocytes at the top layer of a Transwell model; (2) 6-aminonicotinamide, which is known to target glycolysis and reduce cell growth in a variety of tumors^40–42^, including MCF-7 breast cancer cells^41^, using similar or higher drug concentrations (50 µM, 100 µM and dimethylsulfoxide (DMSO) as a control)^41^; and (3) etomoxir, an inhibitor of FAO^43^ that suppresses tumor cell growth^44,45^, including breast cancer^46,47^, using concentrations previously used for MCF-7 cells (100 µM, 200 µM and DMSO as a control)^47^. Using a luminescent cell viability assay (Methods), we observed an increased reduction in the number of cancer cells cultured with CRISPRa-*UCP1*-AAV adipocytes compared to 6-aminonicotinamide and a slightly increased reduction compared to etomoxir (two-tailed *t*-test *P* *=* 0.0163 versus *P* = 0.0319 for CRISPRa-*UCP1*-AAV or etomoxir, respectively; Extended Data Fig. 1n).

### Modulated human adipose organoids suppress xenograft growth

**Extended Data Fig. 2:**
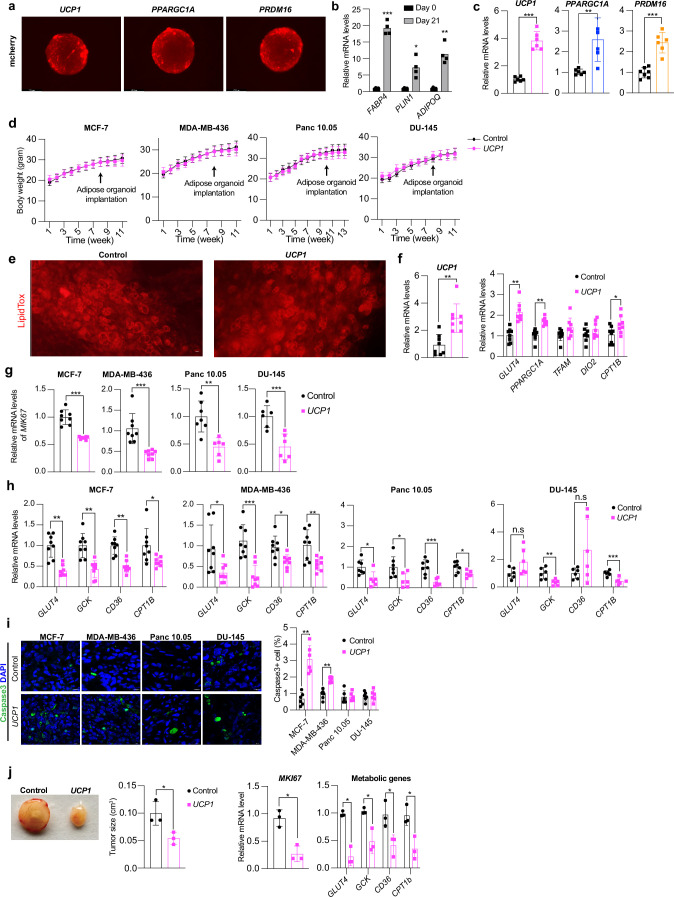
CRISPRa upregulation of *UCP1*, *PPARGC1A*, and *PRDM16* in human adipose organoids and proliferation and metabolic gene expression in tumors co-transplanted with CRISPRa-modulated adipose organoids. **a**, Representative images of human adipose organoids transduced with *UCP1*, *PPARGC1A*, and *PRDM16* AAV9 CRISPRa showing mCherry expression. White scale bar on lower left depicts 176.6μm. **b**, qRT-PCR of *FABP4*, *PLIN1*, and *ADIPOQ* in adipose organoids (n = 4 biological replicates). **c**, qRT-PCR of *UCP1*, *PPARGC1A*, and *PRDM16* in human adipose organoids transduced by AAV9 CRISPRa (n = 5-7 biological replicates). **d**, Body weight of mice co-transplanted with *UCP1*-modulated adipose organoids and cancer cells (n = 8 mice). **e**, Immunofluorescence of LipidTox-stained-adipose organoids after implantation near MCF-7 xenografts. White scale bar on lower right bar depicts 100μm. **f**, qRT-PCR of *UCP1*, *GLUT4, PPARGC1A*, *TFAM, DIO2*, and *CPT1B* of adipose organoids after implantation near MCF-7 xenografts (n = 8 biological replicates). **g**, qRT-PCR of *MKI67* in xenograft tumors derived from MCF-7, MDA-MB-436, Panc 10.05, and DU-145 cancer cells co-transplanted with *UCP1*-CRISPRa modulated adipose organoids (n = 6–8 biological replicates). **h**, qRT-PCR of *GLUT4, GCK, CD36*, and *CPT1B* in xenograft tumors (n = 6–8 biological replicates). **i**, Representative images and quantification of immunofluorescence staining for Caspase 3 in tumors co-implanted with *UCP1*-CRISPRa adipose organoids (n = 6 cryosections). White scale bar on lower right bar depicts 10μm. **j**, Representative images, volume, qRT-PCR of proliferation maker, *MKI67* and metabolic genes, including *GLUT4*, *GCK*, *CD36*, and *CPT1b* of MCF tumors co-implanted with primary adipose organoids (N = 3 mice). All statistical tests were carried out using a two tailed t-test and data are represented as mean ± standard deviation (SD) *≤0.05, **≤0.01, ***≤0.001.

To examine whether CRISPRa-modulated adipocytes could inhibit cancer growth in tumor xenograft models, we co-transplanted four cancer cell lines (MCF-7 and MDA-MB-436 (breast), Panc 10.05 (pancreas) and DU-145 (prostate)) with CRISPRa-modulated human adipose organoids. Although adipocytes could be used for co-transplantation, adipose organoids offer added advantages, such as providing a three-dimensional (3D) culture that can better recapitulate the heterogeneity of adipose tissue, enhanced response to endogenous stimuli and the ability to form tissue microenvironments that could better integrate with cancer cells following transplantation. We initially established culturing conditions for human adipose organoids. We used immortalized human preadipocytes grown using methods that combine features from three different 3D culturing protocols^48,49^. In brief, we cultured human preadipocytes in basal DMEM media supplemented with 10% FBS in Nunc 96-well plates treated with Nunclon Delta. Organoids formed after 48 h and were then differentiated into adipose organoids using a differentiation cocktail containing IBMX, dexamethasone, insulin, T3 and rosiglitazone. Adipocytes formed 21 days post differentiation (Extended Data Fig. 2a). Using RT–qPCR, we analyzed these organoids for various adipogenic markers, including *FABP4*, *PLIN1* and *ADIPOQ*, finding all to be expressed (Extended Data Fig. 2b). We further tested our ability to upregulate *UCP1*, *PPARGC1A* and *PRDM16* in these organoids using similar methods and AAV gRNAs as in the adipocytes, finding all three genes to show significant upregulation of the target genes (Extended Data Fig. 2c). With the establishment of these adipose organoid-culturing and CRISPRa conditions, we next set out to test whether they can suppress xenograft cancer growth.

**Fig. 2:**
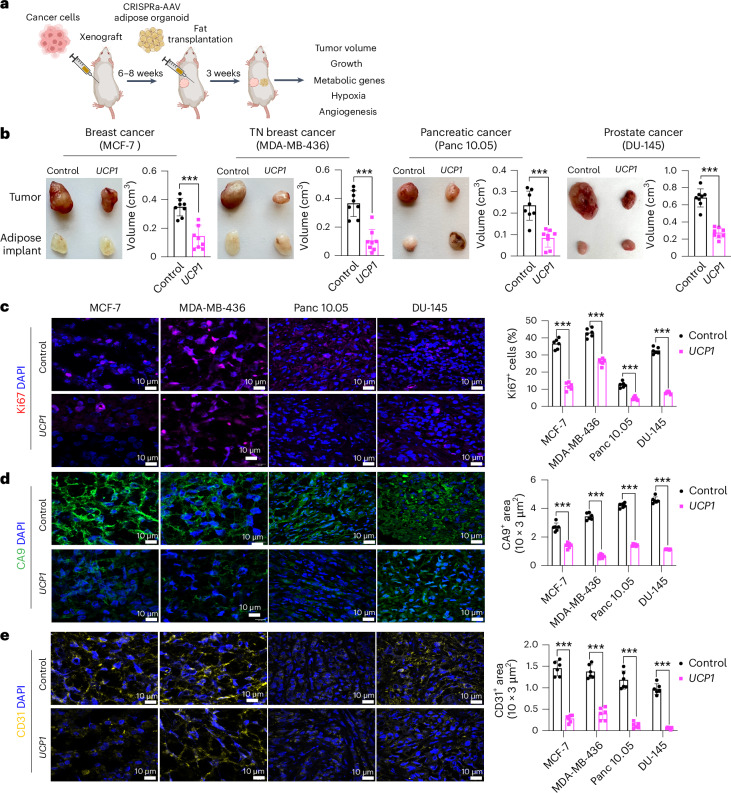
Co-transplantation of xenografts with *UCP1*-CRISPRa-modulated human adipose organoids suppresses tumor growth. **a**, Schematic of the co-transplantation model for xenografts and *UCP1*-CRISPRa-treated human adipose organoids in immune-deficient SCID mice and their subsequent phenotyping (created with BioRender.com). **b**, Representative images of xenograft tumors from various cancer cell lines, including breast (MCF-7 and MDA-MB-436), pancreatic (Panc 10.05) and prostate cancer (DU-145), that were co-transplanted with *UCP1*-CRISPRa human adipose organoids or control (dCas9–VP64 only) adipose organoids (*n* = 8 mice per treatment). TN, triple negative. The bar chart to the right of the images shows the volume of xenograft tumors that were co-transplanted with *UCP1*-CRISPRa human adipose organoids compared to control (dCas9–VP64 only) (*n* = 6–8 mice). **c**–**e**, Immunofluorescence staining and quantification of Ki67 (**c**), CA9 (**d**) and CD31 (**e**) in cryosections of xenograft tumors (*n* = 4–5 sections per treatment). Scale bars, 10 μm. All statistical tests were carried out using a two-tailed *t*-test and data are represented as mean ± s.d. ***P ≤ 0.001.

To generate xenografts, cancer cells were subcutaneously implanted into immuno-compromised SCID mice. After 6–8 weeks, *UCP1*-CRISPRa-treated human adipose organoids were mixed with Matrigel and co-transplanted adjacent to palpable tumors. For all subsequent assays, we only used *UCP1*-CRISPRa, as it showed the most optimal results in our cell culture experiments. Some reports have suggested that brown fat could be linked to cancer-associated cachexia (loss of skeletal muscle and fat)^50^; therefore, each week we measured the body weight of mice co-transplanted with cancer cells and *UCP1*-modulated adipose organoids. We found no significant differences in body weight between CRISPRa-treated and control mice (Extended Data Fig. 2d). Tumors and human adipose organoids were collected after 3 weeks (Fig. 2a). Adipose organoids were stained with LipidTox, showing adipocytes were still present in them 3 weeks after implantation (Extended Data Fig. 2e). These organoids also had increased gene expression levels of *UCP1*, some brown fat genes including *PPARGC1A* and *CPT1b* and the *GLUT4* glucose transporter compared to control adipose organoids (Extended Data Fig. 2f). All tumor types co-transplanted with CRISPRa-modulated human adipose organoids were significantly smaller than dCas9–VP64-only transplanted human adipose organoids, having over 50% reduction in volume (Fig. 2b). Gene analysis showed that tumors co-transplanted with CRISPRa-treated adipose organoids had decreased expression of the proliferation marker gene *MIK67* (Extended Data Fig. 2g) as well as reduced marker gene expression for glycolysis (*GLUT4* (except for DU-145), *GCK*) and FAO (*CD36* (except for DU-145), *CPT1B*) (Extended Data Fig. 2h). Using immunofluorescence, we examined additional tumor marker genes and found that all tumors co-transplanted with *UCP1*-CRISPRa-modulated human adipose organoids had markedly reduced Ki67^+^ cells (Fig. 2c). In addition, cancer cells showed decreased levels of hypoxia, identified by having a lower carbonic anhydrase (CA9^+^) area per image view (Fig. 2d). Furthermore, these tumors exhibited decreased levels of CD31^+^ area per image view, indicating reduced microvessel density and suggesting a corresponding lower metastatic potential (Fig. 2e). We also found higher levels of caspase-3^+^ in both MCF-7 and MDA-MB-436 xenografts co-implanted with *UCP1*-CRISPRa adipose organoids compared to controls, indicating higher apoptotic rates (Extended Data Fig. 2i). To show that this approach can work with another adipocyte cell line, we repeated these experiments for MCF-7 xenografts with *UCP1*-CRISPRa primary adipocytes and found that they also significantly suppressed tumor growth (Extended Data Fig. 2j). Taken together, these results show that *UCP1*-CRISPRa-modulated human adipose organoids significantly reduce glycolysis and FAO, reduce hypoxia and inhibit tumor growth for four different cancer types in vivo.

### Modulated adipose organoids outcompete tumors for nutrients

**Extended Data Fig. 3:**
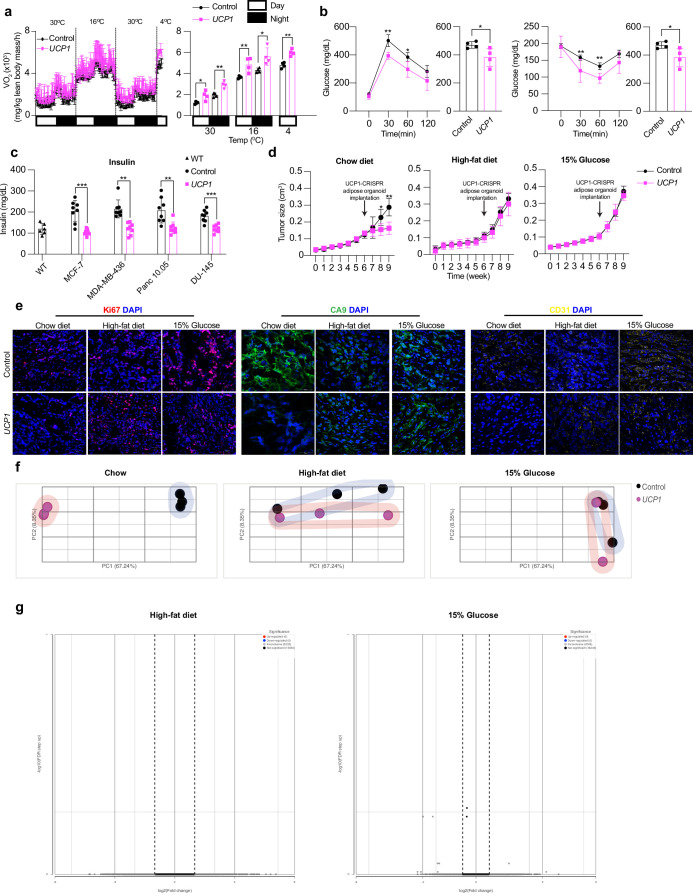
*UCP1*-CRISPRa human adipose organoids increased whole-body energy expenditure, glucose tolerance, and insulin sensitivity. **a**, Whole-body oxygen consumption at 30 °C, 16 °C, and 4 °C during day and night time of mice implanted with *UCP1*-CRISPRa modulated adipose organoids (n = 4 mice). **b**, Glucose tolerance test and insulin tolerance test (n = 4 mice). **c**, Plasma insulin levels of wild type SCID mice (WT) and mice that were co-transplanted with dCas9-VP64 (control) and *UCP1*-CRISPRa adipose organoids and xenografts (n = 6–8 mice). **d**, tumor size of MCF7 tumor xenograft and *UCP1*-CRISPRa treated human adipose organoids in immune-deficient SCID mice fed with standard chow, high-fat diet (HFD), and 15% glucose containing water during 9 weeks (n = 4-5 mice). **e**, Immunofluorescence images from cryosections of xenograft tumors (n = 5 sections per treatment) of Ki67, CA9, and CD31. White scale bar on lower right bar depicts 20μm. **f**, Principle component analysis (PCA) of MCF7 tumors that were co-implanted with *UCP1*-CRISPRa treated human adipose organoids in mice fed with standard chow (left) high-fat diet (middle) or 15% glucose (right); (N = 3). **g**,Volcano plots showing p-value versus fold change of MCF-7 tumors that were co-implanted with *UCP1*-CRISPRa treated human adipose organoids in mice fed with high-fat diet or 15% glucose. No differentially expressed genes were identified, defined as those exhibiting at least a +/− 4.0 fold change, with their expression being significantly different from the basal level (false discovery rate (FDR) adjusted, p < 0.01). All statistical tests in a-d were carried out using a two tailed t-test and data are represented as mean ± standard deviation (SD) *≤0.05, **≤0.01, ***≤0.001.

We next set out to characterize the effect of *UCP1*-CRISPRa adipose organoids on various metabolic parameters and test whether resource competition is involved in the observed cancer suppression. We implanted *UCP1*-CRISPRa or dCas9–VP64 only (negative control) adipose organoids in SCID mice and, after 6 weeks, used the comprehensive lab animal monitoring system (CLAMS) to measure their whole-body oxygen consumption. Mice implanted with *UCP1*-CRISPRa adipose organoids exhibited increased whole-body oxygen consumption at all temperatures (Extended Data Fig. 3a). We also carried out both a glucose and insulin tolerance test on these mice, which revealed that all *UCP1*-CRISPRa-treated mice had increased glucose tolerance and insulin sensitivity (Extended Data Fig. 3b). Following up on these results, we next examined the insulin plasma levels in all four of our xenograft lines, which showed that mice co-transplanted with *UCP1*-CRISPRa adipose organoids had significantly lower insulin levels than those co-transplanted with dCas9–VP64 control adipose organoids and were comparable to levels of wild-type SCID mice (Extended Data Fig. 3c). Combined, these data suggest that *UCP1*-CRISPRa-modulated adipose organoids lead to robust energy consumption, enhanced glucose tolerance and insulin sensitivity and reduced insulin levels.

**Fig. 3:**
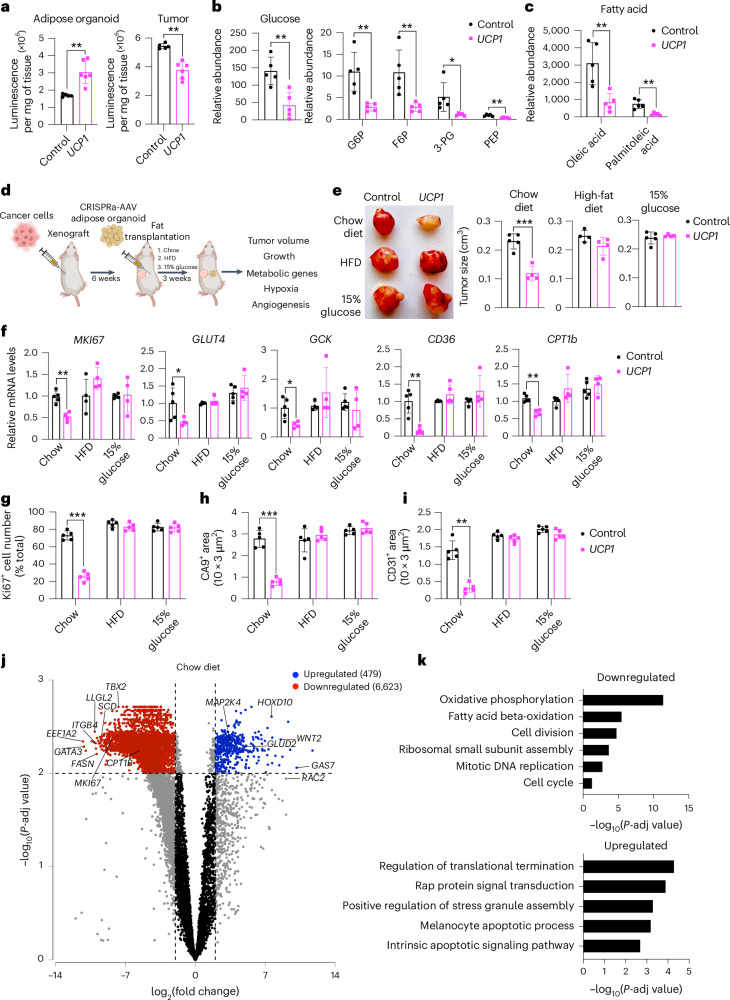
Increasing nutrients reduces *UCP1*-CRISPRa human adipose organoid cancer suppression. **a**, Glucose levels measured from adipose organoids co-transplanted with MCF-7 xenograft tumors using a glucose uptake assay (*n* = 5–6 biological replicates). **b**,**c**, Metabolomics analysis of glucose and glycolysis intermediates, including glucose-6-phosphate (G6P), fructose-6-phosphate (F6P), 3-phosphoglycerate (3-PG) and phosphoenolpyruvate (PEP) (**b**), and fatty acids, including oleic acid and palmitoleic acid (*n* = 5 biological replicates) (**c**). Data are represented as mean ± s.d. **d**, Schematic of the co-transplantation model for MCF-7 tumor xenograft and *UCP1*-CRISPRa-treated human adipose organoids in immune-deficient SCID mice fed with standard chow, HFD or 15% glucose containing water and their subsequent phenotyping (created with BioRender.com). **e**, Representative images and tumor volume of MCF-7 xenografts that were co-transplanted with *UCP1*-CRISPRa human adipose organoids or control (dCas9–VP64 only) from mice on different diets (*n* = 4–5 mice per treatment). **f**, RT–qPCR of proliferation marker gene *MKI67* and metabolic genes (*GLUT4*, *GCK*, *CD36*, *CPT1b*) from MCF-7 xenograft tumors co-transplanted with *UCP1*-CRISPRa or control (dCas9–VP64 only) human adipose organoids in mice fed with various diets (*n* = 4–5 biological replicates). **g**–**i**, Immunofluorescence quantification from cryosections of xenograft tumors (*n* = 5 sections per treatment) of Ki67 (**g**), CA9 (**h**) and CD31 (**i**). **j**, Volcano plot showing *P* value versus fold change of MCF-7 tumors co-implanted with *UCP1*-CRISPRa compared to negative-control-treated human adipose organoids in mice on standard chow diet. Differentially expressed genes are those exhibiting at least a ±fourfold change, with their expression being significantly different from basal level (false discovery rate (FDR)-adjusted *P* < 0.01). **k**, Gene ontology enrichment of significantly downregulated and upregulated genes in MCF-7 tumors from mice on standard chow, using Geneontology.org (https://geneontology.org)^62,63^ with an FDR-adjusted Fisher’s Exact test *P* value of <0.0001. Cell cycle is represented by the term ‘Cell cycle: positive regulation of G2/M transition of mitotic cell cycle’, and cell division by the term ‘Cell division: cytokinesis’. All statistical tests in **a**–**i** were carried out using a two-tailed *t*-test and data are represented as mean ± s.d. *P ≤ 0.05; **P ≤ 0.01; ***P ≤ 0.001.

To examine whether *UCP1*-modulated adipose organoids can prevent glucose uptake in the co-transplanted xenograft tumor, we measure glucose levels in both the adipose organoids and co-implanted MCF-7 tumor. We found that *UCP1*-modulated adipose organoids had higher glucose levels than controls and that xenograft tumors co-implanted with *UCP1*-modulated adipose organoids had lower levels of glucose than control tumors (Fig. 3a and Supplementary Table 1). Metabolomics analysis of these tumors found that glucose levels and glycolytic intermediates, including glucose-6-phosphate, fructose-6-phosphate, 3-phosphoglycerate and phosphoenolpyruvate, were lower in the tumors co-implanted with *UCP1*-modulated adipose organoids than in the control tumors (Fig. 3b and Supplementary Table 1). In addition, the tumors co-implanted with *UCP1*-modulated adipose organoids had lower fatty acid levels, including oleic acid and palmitoleic acid, than the control tumors (Fig. 3c and Supplementary Table 1).

To further test whether nutrient competition is contributory to the ability of *UCP1*-CRISPRa-treated adipose organoids to suppress tumor growth, we performed similar MCF-7 xenograft experiments with mice fed with either standard chow, a high-fat diet (HFD) or 15% glucose containing water (Fig. 3d). We found that tumors co-implanted with *UCP1*-CRISPRa adipose organoids from mice fed with standard chow had significantly lower volume than mice co-implanted with control (dCas9–VP64 only) (Fig. 3e and Extended Data Fig. 3c), which showed significant reduction in volume starting 2 weeks post implantation (Extended Data Fig. 3d). By contrast, HFD-treated or 15% glucose-treated mice showed no apparent difference in tumor growth compared to the negative control (Fig. 3e). Analysis of tumors from mice on a HFD or 15% glucose found that they had expression patterns of the *MKI67* proliferation marker, glycolysis (*GLUT4*, *GCK*) and FAO (*CD36*, *CPT1B*) that are similar to the negative control, whereas mice on a regular chow diet had significantly lower expression for all these markers (Fig. 3f). Similarly, tumors from mice fed with standard chow displayed reduced levels of Ki67^+^ cells, CA9^+^ area, and CD31^+^ area compared to controls, whereas there was no apparent difference in tumors from HFD-treated or 15% glucose-treated mice (Fig. 3g–i and Extended Data Fig. 3e).

We next performed RNA-seq on tumors from all conditions (standard chow, HFD, 15% glucose and negative controls). Principal component analysis revealed that tumors co-implanted with *UCP1*-CRISPRa-modulated adipose organoids in mice fed with a standard chow diet were significantly different than controls, whereas there was no consistent difference in tumors co-implanted with *UCP1*-CRISPRa-modulated adipose organoids and control tumors in mice fed with HFD or 15% glucose water (Extended Data Fig. 3f). Tumors co-implanted with *UCP1*-CRISPRa-modulated adipose organoids in mice fed with a standard chow diet had significantly more global gene expression changes (7,102 differentially expressed genes with 6,623 downregulated and 479 upregulated genes) (Fig. 3j) compared to zero genes for mice treated with a HFD or 15% glucose water (Extended Data Fig. 3g). Among the downregulated genes in the standard chow tumors co-implanted with *UCP1*-CRISPRa-modulated adipose organoids, we found many metabolic genes, including *CPT1b*, *SCD* (stearoyl-CoA desaturase) and *FASN* (fatty acid synthase), and cancer growth and progression regulated genes, such as *MKI67*, *ITGB4* (ref. ^51^), *LLGL2* (ref. ^52^), *GATA3* (ref. ^53^), *EEF1A2* (refs. ^54,55^) and *TBX2* (ref. ^56^) (Fig. 3j). Upregulated genes included tumor suppressor genes, such as *HOXD10* (ref. ^57^), *GAS7* (ref. ^58^) and *MAP2K4* (ref. ^59^), and cancer progression genes, including *WNT2* (ref. ^60^) and *RAC2* (ref. ^61^) (Fig. 3j). We next performed gene ontology enrichment analysis using Geneontology.org (https://geneontology.org/)^62,63^ on differentially expressed genes in tumors of mice fed with standard chow and found downregulated differentially expressed genes to be enriched in pathways involved in metabolic regulation, including FAO, oxidative phosphorylation, cell growth, DNA replication and cell division (Fig. 3k, top). Upregulated enriched pathways included those involved in cellular response to stress response and cell apoptosis (Fig. 3k, bottom). We found no significant pathway changes in tumors in mice fed with HFD or 15% glucose water. In summary, these experiments suggest that *UCP1*-CRISPRa-modulated adipose organoids outcompete tumors for glucose and fatty acids, as increasing fatty acid or glucose levels abolished cancer suppression.

### AMT suppresses cancer development in genetic mouse models

**Extended Data Fig. 4:**
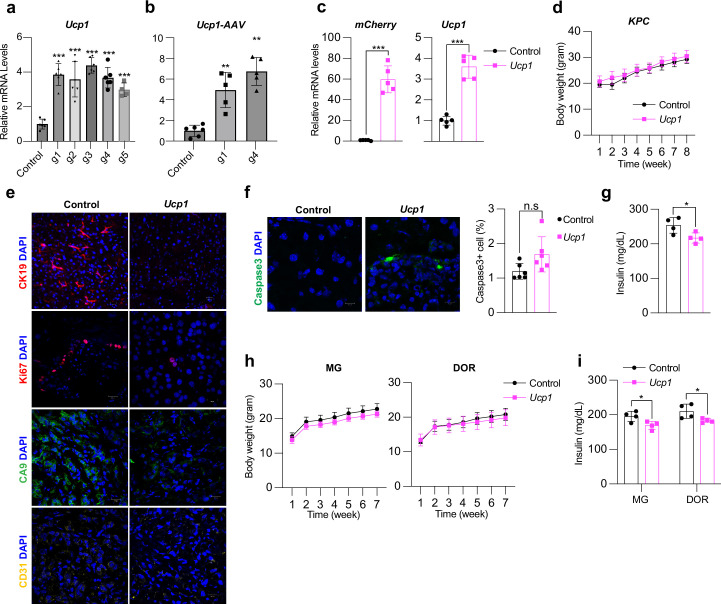
CRISPRa upregulation of *Ucp1* in mouse white adipocytes and phenotypic analysis of mouse genetic cancer models. **a**, qRT-PCR of *Ucp1*, in white adipocytes transfected with CRISPRa using five different gRNAs (n = 4–6 biological replicates). **b**, qRT-PCR of *Ucp1* in mouse adipocytes transduced by AAV9-CRISPRa with two gRNAs (n = 5-6 biological replicates). **c**, qRT-PCR of mCherry and *Ucp1* in mouse adipose organoids (n = 5 biological replicates). **d**, Body weight of pancreatic cancer KPC mice implanted with control (dCas9-VP64) or *Ucp1*-upregulated mouse adipose organoids (n = 5-6 mice). **e**, Immunofluorescence staining of CK19, Ki67, CA9, and CD31 in cryosections of pancreatic tumors (N = 6 sections). White scale bar on lower right bar depicts 20μm. **f**, Representative images and quantification of Caspase3 in cryosections of pancreatic tumors (N = 6 sections). White scale bar on lower right bar depicts 10μm. **g**, Plasma insulin levels of pancreatic cancer genetic mice implanted with either *Ucp1*-CRISPRa adipose organoids or control organoids (n = 4 mice). **h**, Body weight of breast cancer (*MMTV-PyMT*) mice implanted with control (dCas9-VP64) or *Ucp1*-CRISPRa mouse adipose organoids nearby mammary gland (MG) or distal (DOR)(n = 4 mice). **i**, Plasma insulin levels of breast cancer genetic mice implanted with either *Ucp1*-CRISPRa adipose organoids or control organoids nearby mammary gland (MG) or distal (DOR)(n = 4 mice). All statistical tests were carried out using a two tailed t-test and data are represented as mean ± standard deviation (SD) *≤0.05, **≤0.01, ***≤0.001.

To examine whether our AMT approach can prevent cancer development, we used pancreatic and breast cancer genetic mouse models. For pancreatic cancer, we used the KPC mouse model that, upon tamoxifen treatment, develops PDA caused by conditional mutations in *Kras* and *Trp53* (ref. ^64^). For breast cancer, we used *MMTV-PyMT* mice on an FVB background, containing a mouse mammary tumor virus (MMTV) long terminal repeat upstream of the polyomavirus middle T antigen (PyVmT); these mice develop mammary tumors with a mean latency of 53 days^65^. We first designed five gRNAs targeting the mouse *Ucp1* gene and transfected them into mouse 3T3-L1 adipocytes, finding all five to upregulate *Ucp1* (Extended Data Fig. 4a). We next selected two gRNA for AAV9 packaging and infected 3T3-L1 differentiated adipocytes with these viruses along with AAV9 dCas9–VP64, finding that all of them significantly increased *Ucp1* gene expression levels compared to dCas9–VP64-only control (Extended Data Fig. 4b). We used the top-activating gRNA AAV9 for all subsequent experiments. As organoids have several of the aforementioned advantages, we generated adipose organoids using mouse preadipocytes, using techniques similar to those described for human adipose organoids (Methods). We then infected them with AAV9 dCas9–VP64 and *Ucp1*-gRNA and observed both *mCherry* expression from our gRNA virus and significant *Ucp1* upregulation (Extended Data Fig. 4c).

**Fig. 4:**
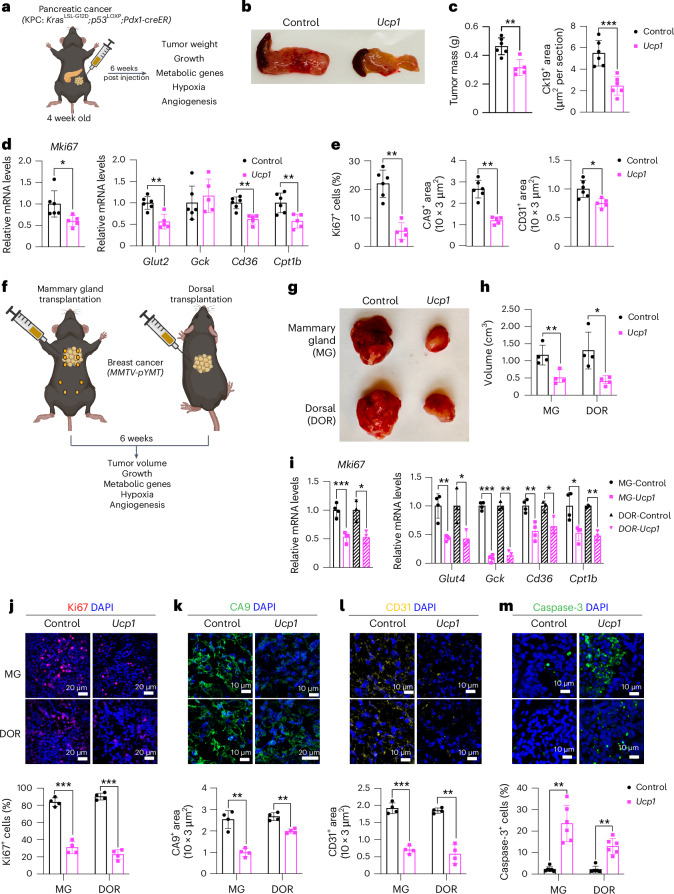
Implantation of *Ucp1*-CRISPRa adipose organoids in pancreatic and breast cancer genetic mouse models suppresses cancer development. **a**, Schematic of the transplantation model for *Ucp1*-CRISPRa-treated mouse adipose organoids in KPC pancreatic cancer mice and their subsequent phenotyping (created with BioRender.com). **b**, Representative images of the pancreas implanted with *Ucp1*-CRISPRa or control (dCas9–VP64 only) mouse adipose organoids (*n* = 5–6 mice per treatment). **c**, Mass and immunofluorescence staining of Ck19 (µm^2^ per section) of the pancreas transplanted with *Ucp1*-CRISPRa-modulated mouse adipose organoids compared to control (dCas9–VP64 only) (*n* = 5–6 mice). **d**, RT–qPCR of the proliferation marker gene *Mki67* and metabolic genes *Glut2*, *Gck, Cd36* and *Cpt1b* from pancreatic tumors co-transplanted with *Ucp1*-CRISPRa-modulated adipocytes (*n* = 5–6 biological replicates). **e**, Immunofluorescence quantification of Ki67, CA9 and CD31 in cryosections of tumors (*n* = 5–6 sections per treatment). **f**, Schematic of the transplantation model for *Ucp1*-CRISPRa-treated mouse adipose organoids in the mammary gland or on the back of *MMTV-PyMT* breast cancer mice and their subsequent phenotyping (created with BioRender.com). **g**, Representative images of the breast tumors that were implanted with *Ucp1*-CRISPRa or control (dCas9–VP64 only) adipose organoids in the mammary gland or on the back of the mice (dorsal) (*n* = 4 mice per treatment). **h**, Volume of the tumors transplanted with *Ucp1*-CRISPRa adipose organoids compared to control (dCas9–VP64 only) (*n* = 4 mice). **i**, RT–qPCR of the proliferation marker gene *Mki67* and metabolic genes *Glut4*, *Gck, Cd36* and *Cpt1b* from breast tumors co-transplanted with *Ucp1*-CRISPRa-modulated adipocytes (*n* = 3–4 biological replicates). **j**–**m**, Immunofluorescence staining and quantification of Ki67 (**j**), CA9 (**k**), CD31 (**l**) and caspase-3 (**m**) in tumor cryosections (*n* = 4–5 sections per treatment). White scale bars on the bottom right represent 10 μm (CA9, CD31, caspase 3) or 20 μm (Ki67 and CA9 DOR-*Ucp1*). All statistical tests were carried out using a two-tailed *t*-test and data are represented as mean ± s.d. *P ≤ 0.05; **P ≤ 0.01; ***P ≤ 0.001.

We next set out to test whether we can suppress pancreatic cancer development using KPC mice. KPC mice were treated with tamoxifen on postnatal day 0–4. At 4 weeks of age, *Ucp1*-CRISPRa or dCas9–VP64-only (negative control) adipose organoids were orthotopically implanted next to the pancreas (Fig. 4a). After 6 weeks, the pancreases were removed and analyzed. Over those 6 weeks, we observed no significant difference in body weight between mice implanted with *Ucp1*-CRISPRa organoids or control organoids (dCas9–VP64 only) (Extended Data Fig. 4d). We found that KPC mice implanted with *Ucp1*-CRISPRa-upregulated adipose organoids had significantly smaller tumors than those in control dCas9–VP64-implanted mice (Fig. 4b), reduced pancreatic mass and Ck19^+^ staining (Fig. 4c and Extended Data Fig. 4e). They also showed reduced expression of the proliferation marker *Mki67* as well as genes involved in FAO (*Cd36*, *Cpt1b*) (Fig. 4d). In addition, we found *Ucp1*-CRISPRa mice to have lower expression levels of the pancreas’ main glucose transporter, *Glut2*, but no significant change for the *Gck* glycolysis marker (Fig. 4d). *Ucp1*-CRISPRa mice also had a lower number of Ki67^+^ cells than control mice as determined by immunofluorescence (Fig. 4e and Extended Data Fig. 4e). In addition, we observed lower CA9^+^ and CD31^+^ area per image view in mouse tumors implanted with *Ucp1*-CRISPR adipose organoids, suggesting reduced hypoxia and angiogenesis (Fig. 4e and Extended Data Fig. 4e). These tumors also had slightly increased levels of caspase-3^+^ cells compared to controls (Extended Data Fig. 4f), suggesting increased apoptosis. Furthermore, we found *Ucp1*-CRISPRa adipose organoids of KPC mice to have significantly reduced insulin levels compared to control mice (Extended Data Fig. 4g).

To examine whether our treatment might provide systematic therapeutic effect in suppressing breast cancer growth, we next used *MMTV-PyMT* female mice, implanting *Ucp1*-CRISPRa or dCas9–VP64 adipose organoids near the third nipples of 4-week-old mice (Fig. 4f). Given that our experiments suggest a nutrient competition model and cold treatment was previously shown to cause widespread BAT activation and subsequent cancer suppression^15^, we also examined whether distal implantation of adipose organoids could suppress cancer development by implanting organoids in the back of 4-week-old mice. At 6 weeks post implantation, tumors were dissected and analyzed. We found no difference in body weight of mice implanted with *Ucp1*-CRISPRa organoids compared to those implanted with control organoids (Extended Data Fig. 4h). Remarkably, we found that both strategies of organoid implantation resulted in significantly reduced tumor size (Fig. 4g) and volume (Fig. 4h) regardless of the site of implantation. Tumors also had decreased expression of the *Mki67* proliferation marker and metabolic genes *Glut4*, *Gck*, *Cd36* and *Cpt1b* (Fig. 4i). The tumors of mice implanted with *Ucp1*-CRISPRa adipose organoids had fewer Ki67^+^ cells than control mice (Fig. 4j). These tumors also had lower CA9^+^ and CD31^+^ area per image view and increased caspase-3^+^ (Fig. 4k–m), suggesting that they have reduced hypoxia and angiogenesis and increased apoptosis. Additionally, mice implanted with *Ucp1*-CRISPRa adipose organoids had significantly lower plasma insulin levels than control mice (Extended Data Fig. 4i). Taken together, our results indicate that our CRISPRa-modulated adipose organoids could have systematic therapeutic effects in suppressing cancer growth.

### Breast dissected modulated adipocytes suppress tumor growth

**Fig. 5:**
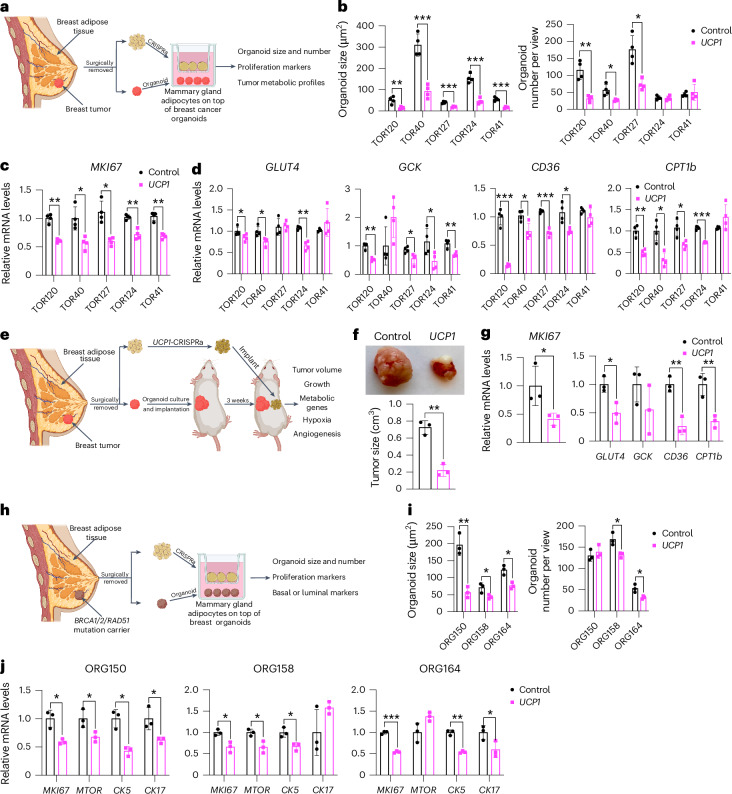
Cancer organoids co-cultured with *UCP1*-CRISPRa adipocytes, both from dissected breast tissue, lead to tumor suppression and prevent cancer development. **a**, Schematic of the co-culturing model of *UCP1*-CRISPRa-modulated human mammary adipocytes and breast cancer organoids from dissected breast tumors (created with BioRender.com). **b**, Cancer organoid size and numbers of breast tumor organoids from five dissected breast tumors that were co-cultured with *UCP1*-CRISPRa adipocytes or control (dCas9–VP64 only) adipocytes (*n* = 4 biological replicates). **c**,**d**, RT–qPCR of the proliferation marker gene *MKI67* (**c**) and metabolic genes *GLUT4*, *GCK, CD36* and *CPT1b* (**d**) of breast cancer organoids that were co-cultured with CRISPRa-modulated adipocytes (*n* = 4 biological replicates). **e**, Schematic of the co-transplantation model for breast cancer organoids and *UCP1*-CRISPRa-treated breast adipocytes in immune-deficient SCID mice and their subsequent phenotyping (created with BioRender.com). **f**, Representative images and tumor size of breast cancer organoids co-implanted with *UCP1*-CRISPRa or control (dCas9–VP64 only) breast adipocytes (*n* = 3 biological replicates). **g**, RT–qPCR of the proliferation marker gene *MKI67* and metabolic genes *GLUT4*, *GCK, CD36* and *CPT1b* of breast cancer organoids that were co-cultured with *UCP1*-CRISPRa or dCas9–VP64-treated breast adipocytes (*n* = 3 biological replicates). **h**, Schematic of the co-culturing model of *UCP1*-CRISPRa-modulated human mammary adipocytes and breast organoids cultured from breast tissues of *BRCA1*/*BRCA2/RAD51D* mutation carrier donors (created with BioRender.com). **i**, Organoid size and numbers of breast organoids from three resected breast tissues that were co-cultured with *UCP1*-CRISPRa adipocytes or control (dCas9–VP64 only) adipocytes (*n* = 3 biological replicates). **j**, RT–qPCR of the proliferation marker genes *MKI67* and *MTOR* and *CK5* and *CK17* of breast organoids that were co-cultured with CRISPRa-modulated adipocytes (*n* = 3 biological replicates). All statistical tests were carried out using a two-tailed *t*-test and data are represented as mean ± s.d. *P ≤ 0.05; **P ≤ 0.01; ***P ≤ 0.001.

**Extended Data Fig. 5:**
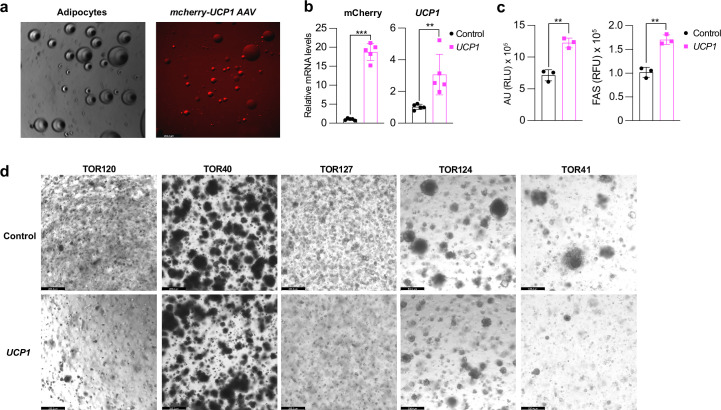
CRISPRa upregulation of *UCP1* in human mammary gland adipocytes. **a**, Representative images of adipocytes isolated from mammary glands that were transduced with *UCP1*-CRISPRa AAV9. Scale bar on lower left depicts 265.2μm. **b**, qRT-PCR of *mcherry* and *UCP1* in CRISPRa-modulated mammary gland adipocytes (n = 5 biological replicates). **c**, Glucose uptake and fatty acid uptake of *UCP1*-CRISPRa adipocytes of sample TOR40 (n = 3 biological replicates). **d**, Bright-field images of adipose organoids that were co-cultured with *UCP1*-CRISPRa modulated mammary gland adipocytes. Scale bars at bottom left depicts 265.2μm (TOR40, TOR120, TOR127) and 530.4μm (TOR41, TOR124). All statistical tests were carried out using a two tailed t-test and data are represented as mean ± standard deviation (SD) **≤0.01, ***≤0.001.

To further demonstrate the therapeutic potential of AMT, we treated adipocytes obtained from dissected human breast tissues with *UCP1*-CRISPRa AAV9 and tested their ability to suppress tumor progression by co-culturing them with breast cancer organoids generated from dissected breast tumors or grown from metastatic pleural effusions (Fig. 5a). Samples were obtained from patients who underwent breast surgery or thoracentesis. For the co-culture experiments, we used five different breast cancer organoids generated from patients with early stage or metastatic triple-negative breast cancer (ER^−^, PR^−^ and human epidermal growth factor receptor 2-negative (HER2^−^)) or hormone receptor-positive (ER^+^ and/or PR^+^), HER2^−^ breast cancer (Supplementary Table 2). Organoids from breast tumor tissue were generated by digesting cells for 1 h in collagenase and organoids from metastatic pleural effusions were generated by isolating tumor spheroids by centrifugation before, in both cases, embedding cancer cells in organoid culture using established protocols that enable long-term propagation of tumor organoids^66^. In parallel, primary human adipocytes were isolated from human breast tissue using an established protocol^67^. For the triple-negative breast cancer organoids derived from primary tumors, we used two different cases, including one in which we generated organoids and isolated mammary gland adipose tissue from the same individual (TOR41). Adipocytes were infected with dCas9–VP64 only (negative control) or *UCP1*-CRISPRa AAV9. After 5 days, they showed strong *mCherry* expression, a fluorescent marker that is part of the gRNA AAV9 (Extended Data Fig. 5a), suggesting that they can be readily infected by our AAVs. In addition, gene analysis showed that both *mCherry* and *UCP1* expression levels were significantly higher in the *UCP1*-CRISPRa-treated adipose organoids than in the control organoids (Extended Data Fig. 5b). Owing to the limited amount of obtainable dissected tissues, we measured glucose and fatty acid uptake of adipocytes isolated from one sample for which we had more abundant material (TOR40). Upon insulin treatment, we found that *UCP1*-CRISPRa-modulated mammary gland adipocytes exhibited increased glucose and fatty acid uptake compared to dCas9–VP64-treated adipocytes (Extended Data Fig. 5c).

To test whether *UCP1*-CRISPRa AAV9 adipocytes can suppress cancer growth, we added them to a hydrogel dome on top of the breast cancer organoids with a fully defined organoid medium^66^ and co-cultured for 5 days (Fig. 5a). In all five cases, we observed a significant reduction in cancer organoid size and, in most organoids, also a reduction in organoid number (TOR124 and TOR41 were not significant) upon incubation with adipocytes infected with *UCP1*-CRISPRa AAV9 compared to the dCas9–VP64-only treated cells (Fig. 5b and Extended Data Fig. 5d). All five cancer organoid cases co-cultured with *UCP1*-CRISPRa had significantly lower proliferation marker *MKI67* expression compared to the negative control (Fig. 5c). We also observed significantly decreased levels of *GLUT4*, *GCK*, *CD36* and *CPT1b* for most of the *UCP1*-CRISPRa co-cultured cancer organoids (Fig. 5d), suggesting that they have lower glycolysis and fatty acid metabolism.

We next tested whether *UCP1*-CRISPRa primary breast adipocytes can suppress breast cancer growth in vivo. We implanted triple-negative breast cancer organoids derived from resected breast tumor into SCID mice for 3 weeks. We used the floating fraction of mature adipocytes in breast tissue. The cells were cultured, treated with *UCP1*-CRISPRa and mixed with Matrigel before their co-implantation for 3 weeks into mice with tumors (Fig. 5e). After 3 weeks, the tumors were dissected and examined. We found that tumors co-implanted with *UCP1*-CRISPRa adipocytes were significantly smaller than those co-implanted with dCas9–VP64-treated adipocytes (Fig. 5f). In addition, tumors co-implanted with *UCP1*-CRISPRa adipocytes had lower expression of *MKI67*, glycolytic genes (*GLUT4* and *GCK*) and FAO genes (*CD36* and *CPT1b*) (Fig. 5g). Overall, our results demonstrate that human adipocytes from dissected breast tissue can upregulate *UCP1* via AAV9-CRISPRa and are able to reduce glycolysis and fatty acid metabolism and suppress breast cancer organoid growth, both in cell culture and xenografts. Furthermore, this work demonstrates the potential clinical utility of an ex vivo autologous transplantation of CRISPRa-modulated adipocytes to treat cancer.

### AMT suppresses high breast cancer risk cell proliferation

To examine whether *UCP1*-CRISPRa mammary adipocytes might prevent cancer development in individuals at high lifetime risk of cancer, we co-cultured them alongside breast organoids from dissected patient-matched breast tissue of *BRCA1*/*BRCA2/RAD51D* mutation carriers (Fig. 5h). We obtained three samples dissected from individual donors carrying a *BRCA1* mutation (ORG158), a *BRCA2* mutation (ORG150) and a *RAD51D* mutation (ORG164) (details of specific mutations and patient history in Supplementary Table 2). In all three cases, we observed that breast organoids co-cultured with donor-matched *UCP1*-CRISPRa adipocytes were significantly smaller and had lower organoid numbers (other than ORG150) than in the negative control (Fig. 5i). In addition, we found that *BRCA1*/*BRCA2* breast organoids co-cultured with *UCP1*-CRISPRa adipose organoids had lower expression levels of known proliferation markers *MKI67* and *MTOR* than that of controls (Fig. 5j). Furthermore, these breast organoids exhibited reduced expression levels of *CK5* and *CK17* (other than ORG158) suggesting reduced basal–luminal phenotypes in these *BRCA1*/*BRCA2/RAD51D-*heterozygous luminal progenitor-predominant breast cells^68,69^ (Fig. 5j). In summary, our results suggest that *UCP1*-CRISPRa breast adipocytes may inhibit premalignant phenotypes in breast organoids from donors with inherited cancer predisposition syndromes.

### Inducible or cell-scaffold AMT suppresses cancer progression

**Fig. 6:**
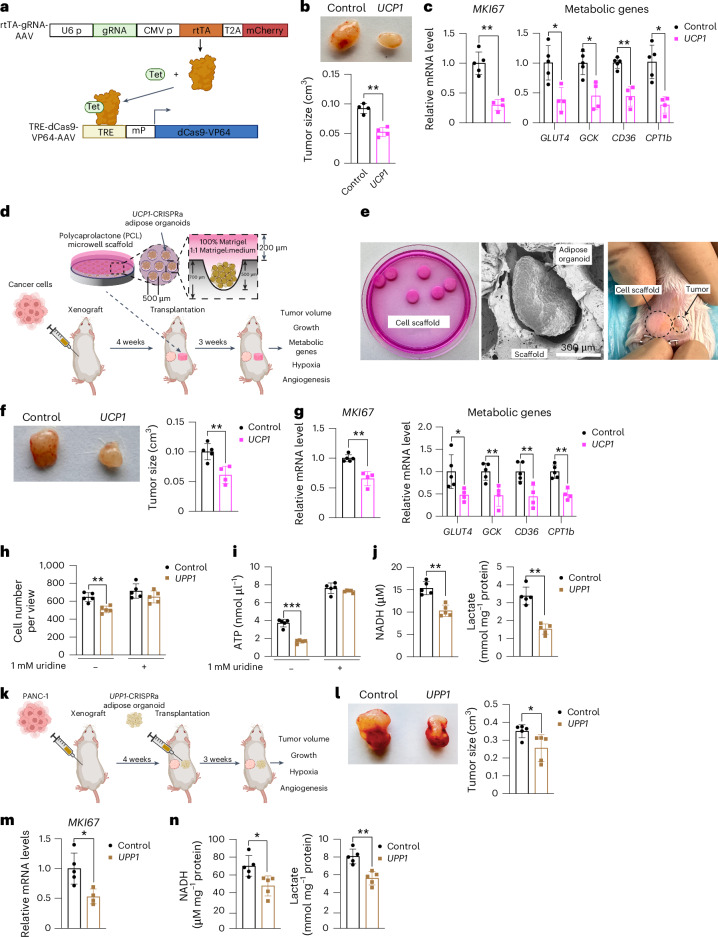
Development of inducible AMT systems and the use of AMT to upregulate *UPP1* to suppress PDA. **a**, Schematic of the inducible CRISPRa-AAV system (created with BioRender.com). Upon tetracycline treatment, rtTA binds to TRE and induces dCas9–VP64 expression. **b**, Representative image and tumor size of MCF-7 tumors that were co-implanted with *UCP1*-CRISPRa or control (dCas9–VP64 only) human adipose organoids (*n* = 4 biological replicates). **c**, RT–qPCR of proliferation marker gene *MKI67* and metabolic genes (*GLUT4*, *GCK*, *CD36* and *CPT1b*) of tumors co-implanted with CRISPRa-modulated adipocytes (*n* = 4–5 biological replicates). **d**, Schematic of co-transplantation of MCF-7 tumors and cell scaffolds containing *UCP1*-CRISPRa or control (dCas9–VP64 only) human adipose organoids and tumor phenotyping (lower panel created with BioRender.com). **e**, Representative images of cell scaffolds in plate (left), electron microscopy image (1.0 kV, ×500 and 7.895 mm lens) of scaffold containing an adipose organoid (middle) and the scaffold implanted in mice (right). **f**, Representative image and tumor size of MCF-7 tumors co-implanted with cell scaffold containing *UCP1*-CRISPRa or control (dCas9–VP64 only) human adipose organoids (*n* = 4–5 biological replicates). **g**, RT–qPCR of the proliferation marker gene *MKI67* and metabolic genes (*GLUT4*, *GCK*, *CD36*, and *CPT1b*) of tumors co-implanted with CRISPRa-modulated adipocytes (*n* = 4–5 biological replicates). **h**, Cell number per view of PANC-1 pancreatic cancer cells that were co-cultured with *UPP1*-CRISPRa or control (dCas9–VP64 only) human adipocytes in media without (–) or with (+) 1 mM uridine (*n* = 5 biological replicates). **i**, ATP levels measured in PANC-1 pancreatic cancer cells co-cultured with *UPP1*-CRISPRa-modulated adipocytes without (–) or with (+) excess uridine (*n* = 5 biological replicates). **j**, NADH and lactate levels in PANC-1 pancreatic cancer cells that were co-cultured with *UPP1*-CRISPRa-modulated adipocytes without the addition of uridine (*n* = 5 biological replicates). **k**, Schematic of the co-transplantation of PANC-1 tumors with *UPP1-*CRISPRa-modulated adipose organoid in SCID mice. **l**, Representative image and size of PANC-1 xenograft tumor co-implanted with dCas9–VP64 or *UPP1*-CRISPRa-modulated adipose organoids (*n* = 5 biological replicates). **m**, RT–qPCR of *MKI67*. **n**, NADH and lactate levels in PANC-1 tumors that were co-implanted with dCas9–VP64 or *UPP1*-CRISPRa-modulated adipose organoids (*n* = 5 biological replicates). All statistical tests were carried out using a two-tailed *t*-test and data are represented as mean ± s.d. *P ≤ 0.05; **P ≤ 0.01; ***P ≤ 0.001.

**Extended Data Fig. 6:**
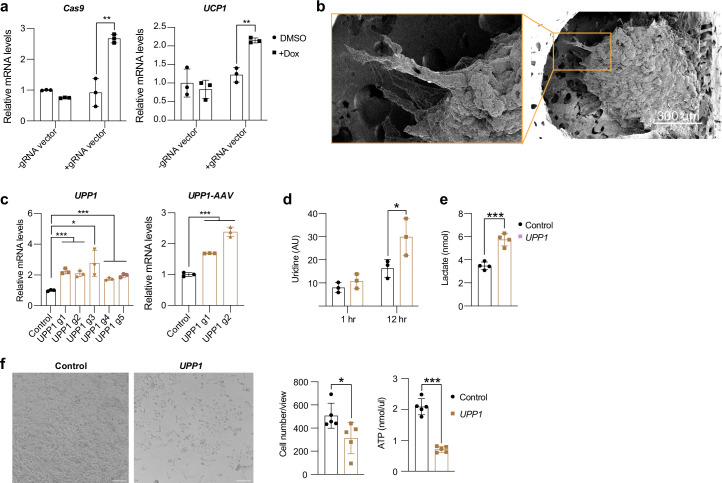
Inducible CRISPRa-modulated adipose organoids and CRISPRa-upregulation of *UPP1* in adipocytes to suppress cancer growth of pancreatic ductal adenocarcinoma. **a**, qRT-PCR of *Cas9* and *UCP1* in adipocytes transduced with inducible CRISPRa AAV and treated with either DMSO or doxycycline (Dox) (n = 3 biological replicates). **b**, Electron microscopy image of adipose organoids implanted in a microwell scaffold showing the organoid attached to the scaffold with filopodia and lamellipodia (1.0 kV, 1500x, and 7.861 mm lens). **c**, qRT-PCR of *UPP1* in white adipocytes transfected with CRISPRa using five different gRNAs (left) and AAV transduced (right) with two gRNAs targeting the *UPP1* promoter (n = 3 biological replicates). **d**, Uridine uptake after 1 or 12 hours measured with incubating *UPP1*-CRISPRa modulated adipocytes with 3H-uridine (n = 3 biological replicates). **e**, Lactate levels of *UPP1*-CRISPRa modulated adipocytes (n = 4 biological replicates). **f**, Representative images, cell numbers/view, and ATP levels of PANC-1 cells that were co-cultured with *UPP1*-modulated adipocytes or control (dCas9-VP64 only) adipocytes in high-glucose media (n = 5 biological replicates). White scale bar on lower right bar depicts 100μm. All statistical tests were carried out using a two tailed t-test and data are represented as mean ± standard deviation (SD) *≤0.05, **≤0.01, ***≤0.001.

To further enhance the translation capabilities of AMT, we developed inducible AAV vectors designed to turn on dCas9–VP64 upon tetracycline treatment, thereby enabling tight regulation of *UCP1* gene expression in adipocytes or adipose organoids. We cloned the reverse tetracycline-control transactivator (rtTA) downstream of the CMV promoter into the gRNA-AAV vector and the tetracycline response element (TRE) and a minimal promoter upstream of dCas9–VP64 in the dCas9–VP64 AAV vector. Upon tetracycline treatment, rtTA binds to TRE and induces expression of dCas9–VP64 (Fig. 6a). To first test this system, we infected TRE-dCas9-VP64-AAV with or without the rtTA-*UCP1*-gRNA-AAV, followed by the addition of DMSO or doxycycline to human adipocytes. We found that only cells in which both TRE-dCas9-VP64-AAV and rtTA*-UCP1*-gRNA-AAV were transduced and treated with doxycycline had increased expression levels of *Cas9* and *UCP1* (Extended Data Fig. 6a). We next generated MCF-7 xenografts in SCID mice and, after 4 weeks, implanted inducible *UCP1*-CRISPRa human adipose organoids. Following adipose organoid implantation, mice were fed a doxycycline diet for 3 weeks and tumors were subsequently dissected and examined. We found that tumors co-implanted with the inducible *UCP1*-CRISPRa human adipose organoids were significantly smaller than ones co-implanted with dCas9–VP64-treated adipose organoids (Fig. 6b). These tumors also had lower expression of the proliferation marker *MKI67*, glycolytic genes (*GLUT4* and *GCK*) and FAO genes (*CD36* and *CPT1b*) (Fig. 6c).

To further showcase the clinical applicability of AMT, we inserted *UCP1*-CRISPRa human adipose organoids into a polymer microwell scaffold generated from polycaprolactone (PCL) and implanted it alongside tumors (Fig. 6d). The microwell scaffold organoid delivery platform is made from biodegradable polyester PCL and manufactured using a microfabrication process involving photolithography and micromolding^70–72^. We made PCL scaffolds patterned with arrays of cylindrical microwells^73–77^. The microwells present a controlled 3D microenvironment that provides a supportive niche for the survival, function and enhanced integration of transplanted organoids^72,78,79^. Here, we manufactured microwells of 500 μm diameter, specifically designed to accommodate and spatially organize adipose organoids for transplantation. Using scanning electron microscopy (SEM), we found that adipose organoids attached to the microwell scaffold with filopodia and lamellipodia, suggesting that the implant is stable and can be extracted after implantation without leaving residual organoids (Extended Data Fig. 6b). We next generated MCF-7 xenografts in SCID mice for 4 weeks, loaded *UCP1*-CRISPRa or dCas9–VP64-only (negative control) human adipose organoids onto the device and co-implanted them next to the xenograft (Fig. 6d,e). After 3 weeks, tumors were dissected and examined. We found that tumors co-implanted with the scaffold containing *UCP1*-CRISPRa human adipose organoids were significantly smaller than tumors co-implanted with the control scaffold adipose organoids containing dCas9–VP64 only (Fig. 6f). Furthermore, these tumors had lower expression levels of the proliferation marker *MKI67*, glycolytic genes (*GLUT4* and *GCK*) and FAO genes (*CD36* and *CPT1b*) (Fig. 6g). Combined, these data show that AMT can be used in an inducible manner or delivered by an integrated cell-scaffold delivery platform that can be readily removed or replaced to fit the changing tumor metabolic landscape.

### Uridine-based AMT suppresses PDA

To examine whether AMT can be used to treat other cancer-associated metabolic pathways, we upregulated the uridine phosphorylase 1 (*UPP1*) gene in human adipocytes and adipose organoids and tested whether they can suppress PDA, which is known to use uridine-derived ribose in glucose-restricted conditions^35^. Using CRISPick^36^, we designed five gRNAs to upregulate *UPP1* gene expression in human adipocytes and found that all gRNAs were able to induce *UPP1* expression in these cells (Extended Data Fig. 6c). We next generated an AAV9 virus for two gRNAs and transduced human adipocytes with AAV9, finding that both gRNAs significantly induced *UPP1* gene expression (Extended Data Fig. 6c). We used the top-activating gRNA AAV for all subsequent experiments. We next transduced human adipocytes with *UPP1*-CRISPRa AAV9 and incubated these cells with ^3^H-labelled uridine. We found these adipocytes to significantly uptake uridine during a 12-h incubation (Extended Data Fig. 6d) and have increased lactate levels, a byproduct of uridine catabolism (Extended Data Fig. 6e). We next co-cultured a PDA cell line, PANC-1, with *UPP1*-CRISPRa or dCas9–VP64 only (negative control) human adipocytes on top of Transwell plates. Cells were cultured in low-glucose-containing media with or without an excess amount of uridine. We found that PANC-1 cells that were co-cultured with the *UPP1*-CRISPRa adipocytes had a significantly lower number of cells than those co-cultured with dCas9–VP64 adipocytes (Fig. 6h). This growth suppression was abolished with the addition of uridine (Fig. 6h). PANC-1 cells that were co-cultured with *UPP1*-CRISPRa adipocytes had lower ATP levels than control PANC-1 cells. By contrast, with an excess amount of uridine, there was no apparent difference in ATP levels between PANC-1 cells with *UPP1*-CRISPRa adipocytes and control cells (Fig. 6i). Furthermore, PANC-1 cells that were co-cultured with *UPP1*-CRISPRa-modulated adipocytes had lower NADH and lactate levels than control cells (Fig. 6j). We next carried out a similar study but grew the cells in a high-glucose-containing media and found that the growth suppression of PANC-1 by *UPP1*-CRISPRa adipocytes was reduced (Extended Data Fig. 6f). Combined, these assays suggest that *UPP1*-CRISPRa adipocytes have increased uridine use and can outcompete PDA for uridine use in low-glucose conditions.

We next set out to test whether *UPP1*-CRISPRa can suppress PDA xenograft growth. We implanted PANC-1 cells in SCID mice for 4 weeks, followed by the implantation of *UPP1*-CRISPRa and dCas9–VP64 only (negative control) adipose organoids (Fig. 6k). After 3 weeks, tumors were dissected and examined. We found that tumors co-implanted with *UPP1*-CRISPRa human adipose organoids were significantly smaller than those co-implanted with dCas9–VP64 adipose organoids (Fig. 6l). In addition, these tumors had lower expression of the proliferation marker *MKI67* (Fig. 6m). All the byproducts of uridine catabolism, including NADH and lactate, were found to be significantly reduced in tumors co-implanted with *UPP1*-CRISPRa human adipose organoids (Fig. 6n). In summary, these results suggest that CRISPRa upregulation of *UPP1* in adipocytes and adipose organoids reduces uridine in the tumor microenvironment of PDA and leads to cancer growth suppression. These results also demonstrate the clinical versatility and capacity of AMT to target various cancer metabolic pathways.

## Discussion

Cancer cells are fast-proliferating cells that require large amounts of nutrients, including glucose and fatty acids. They can reprogram metabolic pathways to use available substrates in the surrounding environment. Targeting their metabolism can be a potent cancer treatment. Here, we developed a cell-based cancer therapeutic approach that uses modified adipocytes to target various cancer metabolic pathways and has the potential to treat a wide variety of cancers. Adipocytes offer a unique ex vivo therapeutic system, with many of the needed procedures already established in the clinic. Liposuction and fat transplantation are commonly used in many surgical procedures, such as aesthetic and reconstructive surgery. Owing to successful engraftment, adipose tissue transplantation has progressively evolved, not only in plastic and reconstructive surgery but also for therapeutic treatments^80^. Several reports using rodent models have shown that BAT transplantation has beneficial metabolic outcomes^81–84^. These also include the use of *UCP1-*CRISPRa modulation in human white preadipocytes to induce browning, followed by their transplantation in mice on a HFD, leading to improved body weight, glucose tolerance and insulin sensitivity^34^. Our work showcases how these ‘brown’ adipocytes and adipose organoids can be used for cancer treatment. In addition, given that adipocytes are ‘metabolic engines’, they can also be engineered to outcompete tumors that use various metabolic pathways, as we show for PDA. Adipocytes are also extensive secretors^85^, and AMT could be used to secrete tumor-targeting factors. The use of cell scaffolds, as done here, could be leveraged by removing and replacing cell scaffolds that contain adipose organoids that target different cancer metabolic pathways, further ‘personalizing’ this therapeutic approach.

There is a growing interest in implementing human adipose tissue grafting by using adipose stem cells or progenitors because of their resistance to trauma and long-term survival following transplantation. One such example is the use of CRISPRa to upregulate the relaxin family peptide receptor 1 (*RXFP1*) gene in adipose-derived stem cells and their transplantation in a diabetes mellitus-induced erectile dysfunction rat model, showing amelioration of the erectile dysfunction phenotype^86^. Organoids could be advantageous for these cells, as they provide a 3D culture that can better recapitulate the heterogeneity of adipose tissue, respond better to endogenous stimuli and form tissue microenvironments that could more efficiently integrate with cancer cells following transplantation. Several groups have successfully grown adipose organoids from mouse adipose stem cells^33,48,49,87^. Here, we were able to culture human adipose organoids from preadipocytes using various conditions from previous studies^33,48,49,87^. These human adipose organoids exhibited mature adipocyte markers, including *FABP4* and *PLIN1*. Finally, in our study, we show that the CRISPRa modulation can significantly reduce tumor size, glycolysis, fatty acid metabolism and uridine use and improve hypoxia and angiogenesis in cancer mouse models. Further development of these organoids and their modulation as well as determination of the number of cells needed to achieve a therapeutic benefit could improve these attributes and their therapeutic use for a wide range of diseases.

Our work builds on the recent observation that activating BAT through cold exposure increases adipocyte glucose uptake and lipid metabolism and significantly inhibits tumor progression^15^. Here, instead of placing tumor models in cold conditions, we took advantage of CRISPRa to increase the gene expression of key BAT regulators, including *UCP1*, *PPARGC1A* and *PRDM16*, to engineer adipocytes to have increased glucose and fatty acid uptake and metabolism and then used them to deplete resources from cancer cells. Similar to the BAT cold activation studies^15^, we observed that high glucose feeding mitigates cancer suppression. In addition, we show that MCF-7 xenograft mice on a HFD have reduced cancer suppression, suggesting that fatty acid competition is also involved in this mechanism. Implanting CRISPRa-modulated adipose organoids distal to the tumors also led to suppression of cancer growth, suggesting that resource competition, similar to BAT cold activation studies^15^, can be carried out distal to the tumor and that complex surgical implantation procedures for tumors with limited access might not be needed for AMT. The observed tumor suppression could also be a result of additional mechanisms. The CRISPRa-modulated adipose organoids could modulate whole-body metabolism. Previous studies have shown that hyperinsulinemia can lead to cancer growth due to insulin being a powerful mitogen and survival factor^88–90^. For example, the administration of dapagliflozin, an SGLT2 inhibitor that lowers blood glucose, and a controlled-release mitochondrial protonophore (CRMP) suppresses cancer growth in mice by reversing hyperinsulinemia^91^. Given that BAT is widely known to reduce whole-body blood glucose and insulin levels in humans^15,83,92^, we reason that the CRISPRa-modulated adipose organoids could also reduce cancer progression by lowering plasma insulin levels. Indeed, our data, using both xenograft and genetic mouse models, show that mice implanted with CRISPRa-modulated adipose organoids exhibit reduced plasma insulin levels compared to the dCas9–VP64 control mice (Extended Data Figs. 3c and 4g,i).

Among BAT-activating genes, *UCP1* showed the most robust effect in terms of cancer suppression. It would be interesting to further develop this AMT approach to upregulate additional genes that could aid in cancer therapy. These could include, for example, upregulation of *GLUT1* and *GLUT4*, which are the main glucose transporters in adipose cells, with *GLUT4* being the most abundant and insulin-responsive^93^; glucose-metabolism-associated genes, such as the transcription factor *FOXO1* (ref. ^94^) and the G-protein coupled receptors GPR40 and GPR120, which have been implicated in improved glucose uptake and insulin resistance^95,96^; *AIFM2*, which promotes glycolysis in BAT^97^; and FAO-associated genes, including, for example, the fatty acid transporter CD36, a key transporter for FAO, CPT1b and the fatty acid breakdown enzyme, ACC1. Finally, as shown in our study with *UPP1*-CRISPRa and PDA, AMT can be customized to fit different cancer metabolic programs. Additional modifications could also be engineered in these adipocytes and adipose organoids, including the use of their endocrine capabilities^98^ to secrete chemotherapeutic drugs or other cancer therapeutic-associated compounds or take advantage of their extracellular vesicles, which are known to have an important role in metabolic regulation^99^. AMT could also be readily implemented with various cancer treatments (surgery, drugs, chemotherapy, radiation and others) that, combined, will increase therapeutic impact.

In this study, we used AAV-based CRISPRa to upregulate genes. CRISPRa has several advantages, including tight regulation owing to the use of the endogenous regulatory machinery^100^ and the ability to simultaneously upregulate multiple genes. However, it is worth noting that both upregulation and delivery could also be carried out using other modalities. For example, gene upregulation could be carried out using zinc fingers, TALENs, generation of specific mutations using regular CRISPR editing or base or prime editing in promoters or enhancers, or standard overexpression using a cDNA mammalian expression construct of the gene of interest. Adipocytes could also be ‘browned’ using various differentiation cocktails^101^, cold activation^15^ or drugs, such as β3 agonists^102,103^, which could also be used to achieve higher competition for nutrients. In addition, various drugs could be used to induce different metabolic pathways. Delivery could be carried out with other viruses, such as lentiviruses that are widely used for chimeric antigen receptor (CAR)-T cell therapy but have a major caveat of genomic integration, or various non-viral nucleic acid delivery vehicles such as nanoparticles^104^ or virus-like particles^105^. Various drugs could also be used to upregulate specific genes in adipocytes in a global manner in cancer patients. In addition, the downregulation of certain genes in adipocytes or adipose organoids using CRISPR inactivation (CRISPRi), short interfering RNA, CRISPR editing or other techniques could also be used for AMT. For example, a recent study used CRISPR to deplete the nuclear receptor interacting protein 1 (*NRIP1*) gene to make ‘brown’ adipocytes that, upon implantation, decreased the adiposity of mice on a HFD^106^.

A link between obesity, excess amount of WAT and cancer development and progression has been established, with nearly 40% of all cancer deaths in the United States being attributed to obesity^107^. There have been numerous mechanisms proposed to explain how WAT is linked to cancer development and progression, including chronic inflammation, hyperinsulinemia, steroid hormones and adipokines^108–114^. In addition, in glucose-rich conditions, cancer cells synthesize de novo fatty acids from intermediates of the glycolysis–TCA cycle (lipogenesis)^115,116^. The synthesized fatty acids are then used to synthesize triglycerides and are stored as lipid droplets in cancer cells. When energy is needed, these lipid droplets undergo lipolysis to release fatty acids, which are subjected to β-oxidation^116–118^. Given that BAT is highly associated with improved glucose tolerance and insulin sensitivity^119^, one could envision a personalized treatment developed for patients with cancer and obesity, whereby AMT is used not only to target cancer and its unique metabolism but also to treat the patients’ metabolic disease. One major hurdle in our approach that needs to be taken into account is cancer-associated cachexia^50^. Although we did not observe weight loss in our mouse models, a longer treatment time could potentially lead to a reduction in body weight, as was shown for *UCP1*-CRISPRa mice on a HFD^34^. Having the ability to control transgene expression in the modified adipocytes and adipose organoids using drugs (such as tetracycline used in our assays) or the ability to remove these cells with the use of an integrated cell-scaffold delivery platform (as also done in our study) could allow the use of this therapeutic approach in a tightly regulated and reversible manner^120–125^. This potential to turn therapeutic intervention on and off holds the key to effectively addressing concerns regarding unforeseen clinical complications, adverse outcomes, changing patient conditions or emerging developments in treatment regimens or tumor metabolic pathways.

In summary, our results provide proof-of-principle results for a cancer therapeutic approach, termed AMT, that can be further developed and personalized for specific cancers and patients. Similar to CAR-T cell therapy, AMT can be readily used in the clinic because cells can be obtained from cancer patients through liposuction or other procedures, engineered and transplanted back into the same individual for therapeutic benefit. The use of adipocytes from dissected breast tissue, as performed in our study, further showcases the clinical utility of such an ex vivo approach. In particular, this could be particularly straightforward for breast cancer, as many mastectomies are followed up by reconstructive surgery with autologous tissue^126^, which could be manipulated before this procedure. Unlike T cells, adipocytes have a lower immune response^34,127^, which could allow more straightforward development of ‘off-the-shelf’ adipocytes or adipose organoids for cancer and other treatments. Their ease of growth in culture, longevity, robustness, lower multiplicity^128^ and endocrine capabilities, along with existing clinical procedures for removal and transplantation, make them a beneficial cell type for cancer and other cellular-based disease therapies.

## Methods

### Human and mouse adipocytes

For adipocyte differentiation, human preadipocytes or mouse 3T3-L1 preadipocytes were used (both kind gifts from H. Sook Sul, UC Berkeley). For human cells, the cell line was immortalized and a single-cell clone was established from primary preadipocytes (Cell Applications, 802S-05A). Cells were cultured to 100% confluency in DMEM, supplemented with 10% FBS, and fresh media was replaced. After 48 h, cells were subjected to adipocyte differentiation by adding a cocktail of IBMX (0.5 M) (Sigma-Aldrich, 410957), dexamethasone (1 µM) (Sigma-Aldrich, D1756) and insulin (10 µg ml^−1^) (Sigma-Aldrich, I9278). The media was replaced every 2 days with insulin-containing DMEM complete media (Fisher Scientific, 11-965-118) during differentiation.

### Cancer cell lines

All cancer cells were acquired from American Type Culture Collection (ATCC). MCF-7 cells (ATCC, HTB-22) were cultured in Eagle’s minimum essential medium (ATCC, 30-2003), supplemented with 10% FBS and 10 µg ml^−1^ human recombinant insulin (Sigma-Aldrich, I9278). MDA-MB-436 cells (ATCC, HTB-130) were cultured in Leibovitz’s L-15 medium (ATCC, 30-2008) with 10 µg ml^−1^ insulin,16 µg ml^−1^ glutathione (Sigma-Aldrich, G6013) and 10% FBS. SW-1417 cells (ATCC, CCL-238) were grown in Leibovitz’s L-15 medium supplemented with 10% FBS. PANC 10.05 cells (ATCC, CRL-2547) were cultured in RPMI-1640 medium (ATCC, 30-2001) with 10 µg ml^−1^ human insulin and 15% FBS. DU-145 cells (ATCC, HTB-81) were cultured in Eagle’s minimum essential medium with 10% FBS. PANC-1 cells (ATCC, CRL-1469) were cultured in DMEM medium (ATCC, 30-2002) supplemented with 10% FBS.

### CRISPRa-AAV in vitro optimization

Five gRNAs targeting the promoter of human *UCP1*, *PPARGC1A*, *PRDM16* and *UPP1* or mouse *Ucp1* were designed using CRISPick^129^ (Supplementary Table 3). These guides were individually cloned into pAAV-U6-sasgRNA-CMV-mCherry-WPREpA^100^ (Addgene, 217015) at the BstXI and XhoI restriction enzyme sites using the In-Fusion (Takara Bio, 638910) cloning method^100^. A total of 5 × 10^5^ human preadipocytes or mouse 3T3-L1 cells were plated onto 12-well plates and then subjected to the adipocyte differentiation protocol after 2 days of confluency. At day 4 of differentiation, cells were transfected with 0.5 µg of dCas9–VP64 and 0.5 µg of gRNA plasmid using X-tremeGene (Sigma-Aldrich, 6366236001) and CombiMag reagent (OZ Biosciences, CM21000) following the manufacturer’s protocol. At day 8 of differentiation, cells were lysed with Trizol, RNA was collected and cDNA and RT–qPCR were performed as described in the ‘RNA isolation and RT–qPCR’ section below. The two gRNAs with the highest upregulation for each gene were packaged into rAAV9 serotype virions. rAAV9 serotype virions were produced by transfecting AAVpro 293T cells (Takara, 632273) with pCMV-sadCas9-VP64 (Addgene, 115790) or pAAV-U6-sasgRNA-CMV-mCherry-WPREpA^100^ along with packaging vectors, including PAAV2/9n (Addgene, 112865) and pHelper vectors using TransIT293 reagent (Mirus, 2700). After 72 h, AAV particles were collected and purified using the AAVpro Cell & Sup. Purification Kit Maxi (Takara, 6676) and quantified by the AAVpro Titration Kit (Takara, 6233). gRNA AAV (1 × 10^6^ multiplicity of infection (MOI)) and dCas9–VP64 AAV (1 × 10^6^ MOI) were used to infect human and mouse differentiated adipocytes. After 5 days, RNA was collected, cDNA was prepared and RT–qPCR was carried out as described below.

### RNA isolation and RT–qPCR

Total RNA from sorted or cultured cells was extracted using Trizol reagent (Thermo Fisher, 15596026). Reverse transcription was performed with 1 μg of total RNA using the qScript cDNA Synthesis Kit (Quantabio, 95047) following the manufacturer’s protocol. RT–qPCR was performed on the QuantStudio 6 Real-Time PCR System (Thermo Fisher) using Sso Fast (Bio-Rad, 1725205). Statistical analysis was performed using ΔΔCt method with *Gapdh* primers as a control (see primer sequences in Supplementary Table 3).

### Seahorse assay

Human preadipocytes (5 × 10^5^ cells per well) were plated in 12-well plates and subjected to the adipocyte differentiation protocol. Adipocytes and cancer cell lines were trypsinized and reseeded in XF96 plates (Agilent, 102905-100) at 2 × 10^4^ cells per well and assayed the next day. On the day of the experiments, the cells were washed two times and maintained in XF base medium (Agilent, 103334) supplemented with either 1 mM sodium pyruvate (Thermo Fisher, 11360070) and 17.5 mM glucose (Sigma-Aldrich, G7021) for the mitochondria stress test or 2 mM glutamine (StemCell technologies, 07100) for the glycolysis test. For FAO tests, cells were washed and incubated with substrate-limiting medium DMEM (Corning, 17-207-CV) supplemented with 0.5 mM glucose, 1 mM GlutaMAX (Thermo Fisher, 35050061), 0.5 mM carnitine (Sigma-Aldrich, C0283) and 1% FBS. Cells were incubated at 37 °C in a non-CO_2_ incubator for 1 h. The assay was performed using a Seahorse XFe96 analyzer (Agilent, XFe96). Oxygen consumption was measured under 1.5 μM oligomycin (Sigma-Aldrich, 75351), 2 μM FCCP (Sigma-Aldrich, C2920) and 0.5 μM Rotenone (Sigma-Aldrich, R8875) and antimycin (Sigma-Aldrich, A8674). The ECAR was measured under 10 mM glucose, 1 µM oligomycin and 50 mM 2-deoxyglucose (Sigma-Aldrich, D8375).

### Glucose uptake assay

Human preadipocytes (3 × 10^5^ cells per well) were plated in 96-well tissue culture plates and subjected to the adipocyte differentiation protocol. The day before the assay, the media was replaced with low serum-free media. On the day of the assay, using glucose uptake-Glo assay (Promega, J1342), cells were incubated with 1 nM insulin (Sigma-Aldrich, I9278) for 1 h before the assay following the manufacturer’s protocol.

### Fatty acid uptake assay

Human preadipocytes (3 × 10^5^ cells per well) were plated in 96-well tissue culture plates and subjected to the adipocyte differentiation protocol or 1 × 10^5^ human mammary gland adipocytes were plated in 96-well plates. On the day of the assay, the media was replaced with serum-free media. Using the fatty acid uptake kit (Sigma-Aldrich, MAK156), cells were incubated with 1 nM insulin (Sigma-Aldrich, I9278) for 30 min before the assay following the manufacturer’s protocol.

### Western blot

To generate a UCP1-positive control, 2 µg of a FLAG-tagged human UCP1 plasmid (Origene, RC218901) was mixed with 200 µl of Opti-MEM (Fisher Scientific, 31985062) and 4 µl of X-tremeGENE HP DNA Transfection Reagent (Roche, XTGHP-RO) and incubated for 15 min. The mixture was added onto AAVpro 293T cells (Takara, 632273) at 6 × 10^5^ per well in a six-well plate and cells were collected 48 h after the transfection. Cells for all conditions were washed twice with DPBS (Sigma-Aldrich, D8537) and dissolved with RIPA buffer (Fisher Scientific, PI89900) containing proteinase inhibitor cocktails and EDTA (Fisher Scientific, PI78440). After shaking for 5 min at 4 °C, cell lysates were centrifuged at 12,000*g* at 4 °C for 10 min to collect the supernatant, and protein concentrations were determined with a BCA protein assay kit (Fisher Scientific, 23227). The cell lysate was mixed with Laemmli SDS sample buffer, reducing (6X) (Fisher Scientific, J61337.AD) to prepare a 1 µg µl^−1^ concentration and placed at 95 °C for 5 min in a programmed heat block. SDS–PAGE was performed on the denatured lysate (10 µg per lane for GAPDH and 40 µg ml^−1^ for other conditions) using Bolt Bis-Tris Mini Protein Gels, 4–12%, 1.0 mm (Fisher Scientific, NW04120BOX) and Bolt MES SDS Running Buffer (Fisher Scientific, B0002). PageRuler Plus Prestained Protein Ladder (Fisher Scientific, 26619) was used as a molecular weight marker. The gels were transferred onto membranes using iBlot2 (Fisher Scientific, IB23002). The membranes were blocked with PVDF Blocking Reagent for Can Get Signal (Toyobo, NYPBR01) for 1 h and treated with primary antibody solutions for hGAPDH and hUCP1(antibody information in Supplementary Table 4) diluted 1,000-fold with Can Get Signal solution 1 (Toyobo, NKB-101) overnight at 4 °C. The membranes were washed twice with DPBS containing 0.02% Tween 20 (Bio-Rad, 1706531) and a secondary antibody solution diluted with Can Get Signal solution 2 was added (Toyobo, NKB-101). After incubation for 1 h at 20 °C room temperature, the membranes were washed twice with DPBS containing 0.02% Tween 20. Antigens were detected with the ECL 2 Western Blotting Substrate detection reagent (Fisher Scientific, PI80196) and imaged on an ImageQuant 800 (Cytiva).

### Co-culturing CRISPRa-modulated adipocytes with cancer cells

A total of 5 × 10^5^ human preadipocytes were plated in 12-well tissue culture plates and subjected to the adipocyte differentiation protocol. On day 2 of differentiation, cells were transduced with gRNA AAV (1 × 10^6^ MOI) and dCas9–VP64 AAV (1 × 10^6^ MOI). After 6 days, cells were collected and replated into the upper well of a 12-well Transwell plate (Corning, 07-200-150) in which 3 × 10^5^ cancer cells had been plated in the lower well 1 day earlier. The cells were cultured in the adipocyte differentiation media (as described previously) and designated media for each cancer cell line (1:1 ratio). For PANC-1, cells were incubated with or without the addition of 1 mM uridine. After 3 days, cancer cells were collected for imaging, cell viability assay or seahorse assay. RNA was collected, cDNA was prepared and RT–qPCR was carried out as previously described. Differential expression was determined using the ΔΔCt method with *GAPDH* primers as control (primer sequences in Supplementary Table 3).

### Luminescent cell viability

MCF-7 cells were grown as mentioned above and treated for 3 days with either CRISPRa-*UCP1*-AAV adipocytes or dCas9–VP64 as a negative control; 6-aminonicotinamide (Sigma-Aldrich, A68203) at 50 µM or 100 µM with DMSO as negative control; and etomoxir (Sigma-Aldrich, E1905) at 100 µM or 200 μM with DMSO as a negative control. The luminescent cell viability assay was performed using the CellTiter-Glo Luminescent Cell Viability Assay (Promega, G7570) following the manufacturer’s protocol on four biological replicates per condition.

### Human and mouse adipose organoid culture

Human or mouse 3T3-L1 preadipocytes (0.5 × 10^6^ cells) were plated in 96-well Nunclon Sphera ULA U-bottom plates (Thermo Fisher, 174929). Organoids formed after 48 h and were then differentiated into adipose organoids using a differentiation cocktail containing IBMX (0.5 M), dexamethasone (1 µM) and insulin (10 µg ml^−1^). Adipocytes formed 21 days post differentiation. Human adipose organoids were then transduced with gRNA AAV (1 × 10^6^ MOI) and dCas9–VP64 AAV (1 × 10^6^ MOI). After 5 days, organoids were collected and mixed with Matrigel (Corning, 354234) and subcutaneously injected into mice (ten organoids per mouse).

### Co-transplantation of CRISPRa-modulated human adipose organoids and cancer cells

All animal studies were carried out in accordance with the University of California San Francisco (UCSF) Institutional Animal Care and Use Committee, protocol number AN197608. Mice were housed in a 12 h:12 h light-to-dark cycle; chow diet (Envigo, 2018S) and water were provided ad libitum. Immune-deficient SCID mice (5 weeks old; JAX, 001303) were anesthetized using isoflurane and subcutaneously injected with 2 × 10^6^–6 × 10^6^ cancer cells, or ~2 × 10^6^ cells after trypsinization of tumor organoids and mixed 1:1 with Matrigel, in PBS. After 6–12 weeks, depending on the cancer cell lines, the mice were subcutaneously injected with CRISPRa-AAV human adipocytes or adipose organoids to a site adjacent to the tumor. After 3–6 weeks, mice were killed and tumors and adipose implants were collected. Tumor size was measured with calipers and tumor volume was determined according to the standard formula (length × width^2^ × 0.52).

### Immunofluorescence

A portion of the isolated tumors was fixed with 1% paraformaldehyde for 2 h and prepared for cryostat sectioning. Each sample was cut into 5 µm-thick sections and blocked with 3% BSA (Miltenyi Biotec, 130-091-376) blocking solution for 30 min. For Ki67 staining, slides were permeabilized with 1% Triton X-100 PBS solution for 15 min and washed with 0.1% Tween 20 PBS solution before blocking. Slides were then incubated with primary antibodies (information and concentrations are listed in Supplementary Table 4) overnight at 4 °C and washed three times with 0.1% Tween20 PBS. The slides were then incubated with secondary antibodies (information and concentrations are listed in Supplementary Table 4) for 60 min at room temperature and then washed three times with 0.1% Tween 20 PBS. Images were obtained using a confocal microscope (Zeiss, LSM880) and analyzed with Fiji^130^.

### Whole-mount staining

Adipose organoids collected after implantation were fixed with 2% paraformaldehyde overnight and then stained with LipidTox Red (Thermo Fisher, H34476) (1:1,000) for 2 h. Images were acquired using a confocal microscope (Zeiss, LSM880).

### Metabolic and thermogenic measurements

Oxymax–CLAMS (Columbus Instruments) was used to measure oxygen consumption and carbon dioxide production of individual mice. Mice were housed individually at 30 °C, 16 °C and 4 °C under a 12 h:12 h light-to-dark cycle. Food and water were available ad libitum. We calculated the respiratory exchange ratio by dividing the volume of CO_2_ produced by the volume of O_2_ consumed.

### RNA-seq analysis

Total RNA was isolated from tumors using the RNeasy Plus kit (Qiagen, 74136). Sequencing libraries were prepared using TruSeq Stranded Total RNA Library Prep Kit (Illumina) and sequenced by Novogene using Illumina NovaSeq 6000. Using Partek Flow (Illumina), reads were trimmed and aligned to the human genome (hg38) using STAR^131^. Differentially expressed genes were annotated using Limma-voom^132^ and were defined as those that were induced at least ±fourfold, and their expression was significantly different from the basal (5% false discovery rate-adjusted *P* < 0.01). We used Geneontology.org (https://geneontology.org/)^62,63^ to determine gene ontology enrichment using biological processes annotations with Fisher’s Exact test and a 5% false discovery rate-adjusted *P* value of <0.0001. RNA-seq data are available under GEO accession number GSE246231 (ref. ^133^).

### CRISPRa-modulated mouse adipose organoid implantation in genetic mouse models

KPC, a tamoxifen-inducible *Kras*^LSL-G12D^; *p53*^LoxP^; *Pdx1-creER* triple-mutant model of tamoxifen-inducible PDA on a C57/BL6 background, was acquired from the Jackson Laboratory (strain no. 032429). To induce Cre recombination, tamoxifen was administered to pups through lactation following oral gavage of the mother with 6 mg tamoxifen (suspended in corn oil) on postnatal days 0, 1, 2 and 4. We orthotopically implanted mouse adipose organoids (ten organoids per mouse) mixed with Matrigel (Corning, 354234) near the pancreas in 4-week-old mice, using a method similar to a previously published protocol^134^, and dissected the pancreas 6 weeks after the implantation. The *MMTV-PyMT* breast cancer mice were acquired from the Jackson Laboratory (strain no. 002374). Female mice were implanted with mouse adipose organoids at 4 weeks of age (ten organoids per mouse) mixed with Matrigel (Corning, 354234) and subcutaneously injected into mice either at the third nipple or on the back. The tumors were collected 6 weeks post implantation.

### Cancer organoids

Organoids were generated from breast tumor tissue or tumors from malignant effusions. Patients provided their consent for specimen collection using IRB-approved tissue collection protocols at the UCSF and the Brigham and Women’s Hospital. Surgical specimens were obtained from the UCSF Medical Center or Brigham and Women’s Hospital on the day of their procedure, viably frozen as tissue pieces or used to generate formalin-fixed, paraffin-embedded sections or organoid cultures. Each tissue was minced using razor blades and digested in a solution containing DMEM/F12 (Gibco, 11330), 2 mM GlutaMAX (Gibco, 35050), 10 mM HEPES (Gibco, 15630), 50 U ml^−1^ penicillin–streptomycin (Gibco, 15070) and 1 mg ml^−1^ collagenase XI (Sigma-Aldrich, C9407). Tissue digestion was performed at 37 °C with constant shaking at 150 rpm for 1–2 h. Cells were then pelleted by centrifugation, further dissociated by sequentially pipetting with 10 ml, 5 ml and 1 ml pipette tips and then recentrifuged. The resulting cell pellet was used directly to establish organoid cultures by embedding in basement membrane extract, allowing this to harden at 37 °C for 20 min to form a hydrogel dome and then overlaying this dome with Type 1 Organoid Medium as previously described^69,135^. All metastatic cancer organoids were derived from malignant pleural effusions that were collected from patients with metastatic breast cancer who were undergoing thoracentesis at the UCSF Medical Center. The fluid samples were placed on ice within 4 h after collection. Organoids were generated by washing malignant effusions with PBS, collecting tumor spheroids by centrifugation and incubating with 3–5 ml RBC lysis buffer (BioLegend, 420301) for 10–15 min when there were visible RBCs, followed by embedding the cell pellet in organoid culture as described above.

### Metabolomics

The tumors were collected and subjected to metabolomics analysis to measure primary metabolites by the University of California Davis West Coast Metabolomics Center. The data were normalized to tumor mass (Supplementary Table 1).

### Mammary gland adipocyte isolation

For adipocyte extraction, mammary gland adipose tissues from excess tissue removed during breast surgeries were washed with PBS three times and mechanically minced into smaller pieces. The tissue mixture was incubated with PBS with 3% BSA and collagenase I (1 mg ml^−1^) for 45 min at 37 °C and then centrifuged; the top lipid layer was then discarded. The remaining mixture was filtered twice through a 200 µm strainer and centrifuged. Mature adipocytes were isolated and washed twice with PBS and then plated in suspension in six-well plates in high-glucose DMEM media supplemented with 10% FBS. For transduction of CRISPRa, *UCP1* AAV9 (1 × 10^6^ MOI) and dCas9–VP64 AAV9 (1 × 10^6^ MOI) were mixed with 20 µl of AdeoMag (OZ Biosciences, AM71000) and adipocytes and incubated for 15 min. The virus and cell mixture was distributed onto a six-well plate placed upon the magnetic plate and incubated for 15 more minutes.

### Co-culturing of cancer organoids and adipocytes

At 5 days post infection of CRISPRa AAV9, adipocytes were placed in a tissue Transwell culture plate (Corning, 07-200-150) of tumor organoids at a 1:1 ratio of adipocyte media and breast cancer organoid media. Cells were incubated for up to 7 days and then examined for adipocyte and tumor phenotypes.

### Co-implantation of cancer organoids and adipocytes

Immune-deficient SCID mice (5 weeks old; JAX, 001303) were anesthetized using isoflurane and subcutaneously injected with 3 × 10^5^ cancer organoids mixed 1:1 with Matrigel in PBS. After 3 weeks, we subcutaneously injected CRISPRa-AAV mammary adipocytes mixed with Matrigel to a site adjacent to the tumor. After another 3 weeks, tumors and adipose implants were dissected. We measured tumor size with calipers and determined tumor volume according to the standard formula (length × width^2^ × 0.52).

### Tet-On CRISPRa

The rtTA was amplified from pSBtet-GP (Addgene, 60495) along with a sequence encoding for T2A and cloned downstream of the *CMV* promoter in the gRNA-AAV vector digested with NheI and AgeI. The TRE and a minimal promoter were amplified and cloned upstream of dCas9–VP64 in the dCas9–VP64 AAV vector digested with XhoI and AgeI. After adipose organoids were infected with Tet-On *UCP1-*CRISPRa and implanted, mice were fed with a doxycycline diet (Bio-Serv, S3888).

### PCL microwell scaffold fabrication

Microwell scaffolds were fabricated using a combination of microfabrication techniques, including photolithography and micromolding. We used PCL (Sigma-Aldrich, 440744), a biocompatible polymer to fabricate microwell scaffolds. We used a polymer blend of 150 mg ml^−1^ PCL (molecular weight, 80K; Sigma-Aldrich, 9016-00-6) and polyethylene glycol (molecular weight, 2.5K; Sigma-Aldrich, 438197) in trifluoroethanol (Sigma-Aldrich, 8.08259). The polymer solution was stirred on the roller bank overnight at room temperature. We then used photolithography to fabricate the SU8 (Microchem/Kayaku, Y111075) microwell array master mold. The microwell mold was then silanized and polydimethylsiloxane (PDMS) (Thermo Fisher, 178442500) and was used to prepare the microwell template. The PDMS template had a micropatterned spatially organized array of dome-like structures called microwells, each 700 μm in height and 500 μm diameter. Micromolding was used to prepare the PCL microwell scaffold using a PDMS microwell template. Two-step spin casting was used to cast the PCL solution on the PDMS microwell template at 100 rpm and 1,000 rpm for 10 s to regulate the height of the microwell; the microwell diameter was defined by the microwells on the template. The PCL-coated template was allowed to dry at room temperature overnight to evaporate the organic solvent, followed by immersion in deionized water. The polyethylene glycol in the PCL polymer blend was allowed to leach out in a deionized water bath for 5 days to introduce the porosity in the microwell PCL scaffold. Subsequently, the microwell scaffolds were washed and stored in deionized water till further use. The size of the microwells on the PCL microwell scaffold was designed to be suitable to host adipose organoids.

### Adipose organoids microwell scaffold transplantation

The microwell scaffold organoid delivery platform was primed for the transplantation of adipose organoids. The microwell scaffolds were thoroughly washed and dried followed by treatment with air plasma for 3 min in aseptic conditions. We then soaked the scaffolds in a solution of adipocyte differentiation media and Matrigel (Corning, 354234) at 4 °C for 24 h. The adipose organoids were then spin-loaded individually in each microwell at 1,000 rpm and 4 °C. The microwell scaffolds loaded with adipose organoids were then layered with a 1:1 ratio of adipocyte differentiation media and Matrigel. The scaffold-organoids system was then cultured at 37 °C at 5% CO_2_ for 24 h. The immunodeficient SCID mice (JAX, 001303) were antecedently prepped with orthotopic MCF-7 tumor. The adipose organoids-loaded microwell organoid delivery scaffolds were then transplanted into the mice adjacent to the orthotopic tumor. After 3 weeks, tumors and microwell scaffold adipose organoid implants were collected. The tumor size was measured with calipers and tumor volume was determined according to the standard formula (length × width^2^ × 0.52).

### Scanning electron microscopy

Adipose organoids on the microwell scaffolds were meticulously prepared for analysis and visualization of their ultrastructural characteristics using SEM. The organoids on the scaffolds were fixed by immersing them in a 2.5% glutaraldehyde (Electron Microscopic Sciences, 16210) solution in a 0.1 M sodium cacodylate (Sigma-Aldrich, C0250) buffer with a pH of 7.2 at 4 °C over 24 h. Subsequently, samples were thoroughly rinsed for 30 min with 0.1 M sodium cacodylate buffer with a pH of 7.4 to remove residual fixative. To enhance structural contrast, post-fixation was performed using a 2% phosphotungstic acid (Sigma-Aldrich, P4006) in 0.1 M sodium cacodylate buffer at 4 °C for 1 h followed by thoroughly rinsing for 30 min with 0.1 M sodium cacodylate buffer with a pH of 7.4. The samples were then dehydrated by immersing for 20 min each in a graded series of ethanol (35%, 50%, 70%, 95%, 100%) solutions, enabling the gradual removal of water content. Critical-point drying over 24 h using hexamethyldisilazane (Sigma-Aldrich, 440191) ensured the complete removal of moisture from the specimens without causing any damage. Finally, a thin iridium coating of ~2 nm thickness was sputter-coated on the samples to enhance conductivity during SEM imaging. This systematic sample preparation method facilitated high-resolution SEM imaging at 1 kV on Phenom Pharos G2 Desktop FEG-SEM (Thermo Fisher Scientific), enabling a comprehensive exploration of the ultrastructural morphology.

### Uridine and uridine triphosphate (UTP) uptake studies

Adipocytes differentiated and treated with or without *UPP1*-CRISPRa were maintained in a 24-well plate using cell culture medium (see ‘Cell culture media’ section). Before the uptake experiments, the culture medium was discarded and the cells were incubated in 1.0 ml of Hanks’ balanced salt solution (HBSS; Gibco, 14025092) for 10–20 min at 37 °C. After this pre-incubation, trace amounts of either ^3^H-uridine (128 nM) or 3H-UTP (240 nM) were introduced to the cells and incubation continued for 1 h or 12 h. Subsequently, the cells were washed twice with ice-cold HBSS. After washing, the cells were lysed using a lysis buffer composed of 0.1 N NaOH and 0.1% SDS. The radioactivity in the lysate was then quantified through liquid scintillation counting.

### Statistical analysis

Statistical analyses were performed using one-way ANOVA or two-tailed *t*-tests and are described in each figure legend. A *P* value of <0.05 was considered statistically significant. The number of mice or replicates used in each experiment are indicated in the figure legends.

### Reporting summary

Further information on research design is available in the Nature Portfolio Reporting Summary linked to this article.

## Online content

Any methods, additional references, Nature Portfolio reporting summaries, source data, extended data, supplementary information, acknowledgements, peer review information; details of author contributions and competing interests; and statements of data and code availability are available at 10.1038/s41587-024-02551-2.

## Supplementary information

Supplementary Information Supplementary Tables 2–4.

Reporting Summary

Supplementary Table 1 Supplementary Table 1.

## Source data

Source Data Extended Data Fig. 1 Source Data Fig. 1. Raw data for western blots. **a**,**b**, Uncropped images of the western blot displayed in Extended Data Fig. 1l. The same blot was cut and stained on the left for GAPDH (**a**) and UCP1 on the right (**b**) because both proteins were close in size (36 kDa for GAPDH and 33 kDa for UCP1). GAPDH was used as a loading marker because of its use also in RT–qPCR. A total of 10 µg per lane GAPDH, 40 µg per lane dCas9–VP64 only (VP64 (neg.)), 40 µg per lane dCas9–VP64 + UCP1-sgRNA (UCP1-CRISPRa) and 10 µg per lane UCP1 overexpression were loaded in their respective lanes. **c**, Additional western blot using the same samples in **a** and **b** showing 40 µg per lane dCas9–VP64 only (VP64 (neg.)), 40 µg per lane dCas9–VP64 + UCP1-sgRNA (UCP1-CRISPRa) and 10 µg per lane UCP1 overexpression. The PageRuler Plus Protein Ladder (Thermo Fisher) was used as a marker in all western blots.

## Data Availability

All data are available upon request. Requests for materials should be directed to the corresponding author. RNA-seq data are available under GEO accession number GSE246231 (ref. ^133^). Source data are provided with this paper.

## References

[1] Lin, X., Xiao, Z., Chen, T., Liang, S. H. & Guo, H. Glucose metabolism on tumor plasticity, diagnosis, and treatment. *Front. Oncol.* **10**, 317 (2020). 32211335 10.3389/fonc.2020.00317 PMC7069415

[2] Hay, N. Reprogramming glucose metabolism in cancer: Can it be exploited for cancer therapy? *Nat. Rev. Cancer* **16**, 635–649 (2016). 27634447 10.1038/nrc.2016.77 PMC5516800

[3] Liberti, M. V. & Locasale, J. W. The Warburg effect: How does it benefit cancer cells? *Trends Biochem. Sci.* **41**, 211–218 (2016). 26778478 10.1016/j.tibs.2015.12.001 PMC4783224

[4] Nagarajan, S. R., Butler, L. M. & Hoy, A. J. The diversity and breadth of cancer cell fatty acid metabolism. *Cancer Metab.* **9**, 2 (2021). 33413672 10.1186/s40170-020-00237-2 PMC7791669

[5] Palm, W. & Thompson, C. B. Nutrient acquisition strategies of mammalian cells. *Nature* **546**, 234–242 (2017). 28593971 10.1038/nature22379 PMC5541675

[6] DeWaal, D. et al. Hexokinase-2 depletion inhibits glycolysis and induces oxidative phosphorylation in hepatocellular carcinoma and sensitizes to metformin. *Nat. Commun.* **9**, 446 (2018). 29386513 10.1038/s41467-017-02733-4 PMC5792493

[7] Chan, D. A. et al. Targeting GLUT1 and the Warburg effect in renal cell carcinoma by chemical synthetic lethality. *Sci. Transl. Med.* **3**, 94ra70 (2011). 21813754 10.1126/scitranslmed.3002394 PMC3683134

[8] Liu, Y. et al. A small-molecule inhibitor of glucose transporter 1 downregulates glycolysis, induces cell-cycle arrest, and inhibits cancer cell growth in vitro and in vivo. *Mol. Cancer Ther.* **11**, 1672–1682 (2012). 22689530 10.1158/1535-7163.MCT-12-0131

[9] Fernandez, L. P., Gomez de Cedron, M. & Ramirez de Molina, A. Alterations of lipid metabolism in cancer: implications in prognosis and treatment. *Front. Oncol.* **10**, 577420 (2020). 33194695 10.3389/fonc.2020.577420 PMC7655926

[10] Khan, W. et al. Lipid metabolism in cancer: a systematic review. *J. Carcinog.* **20**, 4 (2021). 34321955 10.4103/jcar.JCar_15_20 PMC8312377

[11] Wang, W., Bai, L., Li, W. & Cui, J. The lipid metabolic landscape of cancers and new therapeutic perspectives. *Front. Oncol.* **10**, 605154 (2020). 33364199 10.3389/fonc.2020.605154 PMC7753360

[12] Snaebjornsson, M. T., Janaki-Raman, S. & Schulze, A. Greasing the wheels of the cancer machine: the role of lipid metabolism in cancer. *Cell Metab.* **31**, 62–76 (2020). 31813823 10.1016/j.cmet.2019.11.010

[13] Mason, P. et al. SCD1 inhibition causes cancer cell death by depleting mono-unsaturated fatty acids. *PLoS ONE* **7**, e33823 (2012). 22457791 10.1371/journal.pone.0033823 PMC3310881

[14] Guseva, N. V., Rokhlin, O. W., Glover, R. A. & Cohen, M. B. TOFA (5-tetradecyl-oxy-2-furoic acid) reduces fatty acid synthesis, inhibits expression of AR, neuropilin-1 and Mcl-1 and kills prostate cancer cells independent of p53 status. *Cancer Biol. Ther.* **12**, 80–85 (2011). 21525791 10.4161/cbt.12.1.15721

[15] Seki, T. et al. Brown-fat-mediated tumour suppression by cold-altered global metabolism. *Nature* **608**, 421–428 (2022). 35922508 10.1038/s41586-022-05030-3 PMC9365697

[16] Symonds, M. E., Aldiss, P., Pope, M. & Budge, H. Recent advances in our understanding of brown and beige adipose tissue: the good fat that keeps you healthy. *F1000Res* **7**, F1000 (2018). 30079236 10.12688/f1000research.14585.1 PMC6058473

[17] Virtanen, K. A. et al. Functional brown adipose tissue in healthy adults. *N. Engl. J. Med.* **360**, 1518–1525 (2009). 19357407 10.1056/NEJMoa0808949

[18] Cannon, B. & Nedergaard, J. Brown adipose tissue: function and physiological significance. *Physiol. Rev.* **84**, 277–359 (2004). 14715917 10.1152/physrev.00015.2003

[19] Klingenberg, M. Uncoupling protein—a useful energy dissipator. *J. Bioenerg. Biomembr.* **31**, 419–430 (1999). 10653471 10.1023/a:1005440221914

[20] Suzuki, D., Murata, Y. & Oda, S. Changes in *Ucp1*, *D2* (*Dio2*) and *Glut4* (*Slc2a4*) mRNA expression in response to short-term cold exposure in the house musk shrew (*Suncus murinus*). *Exp. Anim.* **56**, 279–288 (2007). 17660682 10.1538/expanim.56.279

[21] Vimaleswaran, K. S., Radha, V., Deepa, R. & Mohan, V. Absence of association of metabolic syndrome with PPARGC1A, PPARG and UCP1 gene polymorphisms in Asian Indians. *Metab. Syndr. Relat. Disord.* **5**, 153–162 (2007). 18370824 10.1089/met.2006.0032

[22] Feldmann, H. M., Golozoubova, V., Cannon, B. & Nedergaard, J. UCP1 ablation induces obesity and abolishes diet-induced thermogenesis in mice exempt from thermal stress by living at thermoneutrality. *Cell Metab.* **9**, 203–209 (2009). 19187776 10.1016/j.cmet.2008.12.014

[23] Tabuchi, C. & Sul, H. S. Corrigendum: signaling pathways regulating thermogenesis. *Front. Endocrinol. (Lausanne)* **12**, 698619 (2021). 34239501 10.3389/fendo.2021.698619 PMC8259581

[24] Yi, D. et al. Zc3h10 acts as a transcription factor and is phosphorylated to activate the thermogenic program. *Cell Rep.* **29**, 2621–2633.e4 (2019). 31775033 10.1016/j.celrep.2019.10.099 PMC6911170

[25] Puigserver, P. & Spiegelman, B. M. Peroxisome proliferator–activated receptor-γ coactivator 1α (PGC-1α): transcriptional coactivator and metabolic regulator. *Endocr. Rev.* **24**, 78–90 (2003). 12588810 10.1210/er.2002-0012

[26] Lin, J. et al. Defects in adaptive energy metabolism with CNS-linked hyperactivity in *PGC-1*α null mice. *Cell* **119**, 121–135 (2004). 15454086 10.1016/j.cell.2004.09.013

[27] Kajimura, S. Promoting brown and beige adipocyte biogenesis through the PRDM16 pathway. *Int. J. Obes. Suppl.* **5**, S11–S14 (2015). 27152168 10.1038/ijosup.2015.4 PMC4850573

[28] Harms, M. J. et al. PRDM16 binds MED1 and controls chromatin architecture to determine a brown fat transcriptional program. *Genes Dev.* **29**, 298–307 (2015). 25644604 10.1101/gad.252734.114 PMC4318146

[29] Ohno, H., Shinoda, K., Spiegelman, B. M. & Kajimura, S. PPARgamma agonists induce a white-to-brown fat conversion through stabilization of PRDM16 protein. *Cell Metab.* **15**, 395–404 (2012). 22405074 10.1016/j.cmet.2012.01.019 PMC3410936

[30] Kajimura, S. et al. Initiation of myoblast to brown fat switch by a PRDM16-C/EBP-β transcriptional complex. *Nature* **460**, 1154–1158 (2009). 19641492 10.1038/nature08262 PMC2754867

[31] Seale, P. et al. PRDM16 controls a brown fat/skeletal muscle switch. *Nature* **454**, 961–967 (2008). 18719582 10.1038/nature07182 PMC2583329

[32] Kajimura, S. et al. Regulation of the brown and white fat gene programs through a PRDM16/CtBP transcriptional complex. *Genes Dev.* **22**, 1397–1409 (2008). 18483224 10.1101/gad.1666108 PMC2377193

[33] Seale, P. et al. Transcriptional control of brown fat determination by PRDM16. *Cell Metab.* **6**, 38–54 (2007). 17618855 10.1016/j.cmet.2007.06.001 PMC2564846

[34] Wang, C. H. et al. CRISPR-engineered human brown-like adipocytes prevent diet-induced obesity and ameliorate metabolic syndrome in mice. *Sci. Transl. Med.* **12**, eaaz8664 (2020). 32848096 10.1126/scitranslmed.aaz8664 PMC7704293

[35] Nwosu, Z. C. et al. Uridine-derived ribose fuels glucose-restricted pancreatic cancer. *Nature* **618**, 151–158 (2023). 37198494 10.1038/s41586-023-06073-w PMC10232363

[36] Kim, H. K. et al. Deep learning improves prediction of CRISPR-Cpf1 guide RNA activity. *Nat. Biotechnol.* **36**, 239–241 (2018). 29431740 10.1038/nbt.4061

[37] Flint, J. & Shenk, T. Viral transactivating proteins. *Annu. Rev. Genet.* **31**, 177–212 (1997). 9442894 10.1146/annurev.genet.31.1.177

[38] Wu, Z., Yang, H. & Colosi, P. Effect of genome size on AAV vector packaging. *Mol. Ther.* **18**, 80–86 (2010). 19904234 10.1038/mt.2009.255 PMC2839202

[39] Bates, R., Huang, W. & Cao, L. Adipose tissue: an emerging target for adeno-associated viral vectors. *Mol. Ther. Methods Clin. Dev.* **19**, 236–249 (2020). 33102616 10.1016/j.omtm.2020.09.009 PMC7566077

[40] Kaushik, N., Kaushik, N. K., Choi, E. H. & Kim, J. H. Blockade of cellular energy metabolism through 6-aminonicotinamide reduces proliferation of non-small lung cancer cells by inducing endoplasmic reticulum stress. *Biology (Basel)* **10**, 1088 (2021). 34827081 10.3390/biology10111088 PMC8614681

[41] Li, Y. et al. Targeting glucose-6-phosphate dehydrogenase by 6-AN induces ROS-mediated autophagic cell death in breast cancer. *FEBS J.* **290**, 763–779 (2023). 36048131 10.1111/febs.16614 PMC10087799

[42] Varshney, R., Dwarakanath, B. & Jain, V. Radiosensitization by 6-aminonicotinamide and 2-deoxy-d-glucose in human cancer cells. *Int. J. Radiat. Biol.* **81**, 397–408 (2005). 16076755 10.1080/09553000500148590

[43] O’Connor, R. S. et al. The CPT1a inhibitor, etomoxir induces severe oxidative stress at commonly used concentrations. *Sci. Rep.* **8**, 6289 (2018). 29674640 10.1038/s41598-018-24676-6 PMC5908836

[44] Shim, J.-K. et al. Etomoxir, a carnitine palmitoyltransferase 1 inhibitor, combined with temozolomide reduces stemness and invasiveness in patient-derived glioblastoma tumorspheres. *Cancer Cell Int.* **22**, 309 (2022). 36221088 10.1186/s12935-022-02731-7 PMC9552483

[45] Lee, C.-K. et al. Tumor metastasis to lymph nodes requires YAP-dependent metabolic adaptation. *Science* **363**, 644–649 (2019). 30733421 10.1126/science.aav0173

[46] Loo, S. Y. et al. Fatty acid oxidation is a druggable gateway regulating cellular plasticity for driving metastasis in breast cancer. *Sci. Adv.* **7**, eabh2443 (2021). 34613780 10.1126/sciadv.abh2443 PMC8494440

[47] Manerba, M. et al. Metabolic activation triggered by cAMP in MCF-7 cells generates lethal vulnerability to combined oxamate/etomoxir. *Biochim. Biophys. Acta Gen. Subj.* **1863**, 1177–1186 (2019). 30981740 10.1016/j.bbagen.2019.04.008

[48] Strobel, H. A., Gerton, G. & Hoying, J. B. Vascularized adipocyte organoid model using isolated human microvessel fragments. *Biofabrication* **13**, 035022 (2021). 10.1088/1758-5090/abe187 33513595

[49] Taylor, J. et al. Generation of immune cell containing adipose organoids for in vitro analysis of immune metabolism. *Sci. Rep.* **10**, 21104 (2020). 33273595 10.1038/s41598-020-78015-9 PMC7713299

[50] Kir, S. & Spiegelman, B. M. Cachexia and brown fat: a burning issue in cancer. *Trends Cancer* **2**, 461–463 (2016). 28459108 10.1016/j.trecan.2016.07.005 PMC5407404

[51] Bierie, B. et al. Integrin-β4 identifies cancer stem cell-enriched populations of partially mesenchymal carcinoma cells. *Proc. Natl Acad. Sci. USA* **114**, E2337–E2346 (2017). 28270621 10.1073/pnas.1618298114 PMC5373369

[52] Saito, Y. et al. LLGL2 rescues nutrient stress by promoting leucine uptake in ER^+^ breast cancer. *Nature* **569**, 275–279 (2019). 30996345 10.1038/s41586-019-1126-2

[53] Takaku, M., Grimm, S. A. & Wade, P. A. GATA3 in breast cancer: Tumor suppressor or oncogene? *Gene Expr.* **16**, 163–168 (2015). 26637396 10.3727/105221615X14399878166113 PMC4758516

[54] Jia, L. et al. EEF1A2 interacts with HSP90AB1 to promote lung adenocarcinoma metastasis via enhancing TGF-β/SMAD signalling. *Br. J. Cancer* **124**, 1301–1311 (2021). 33473168 10.1038/s41416-020-01250-4 PMC8007567

[55] Giudici, F. et al. Elevated levels of eEF1A2 protein expression in triple negative breast cancer relate with poor prognosis. *PLoS ONE* **14**, e0218030 (2019). 31220107 10.1371/journal.pone.0218030 PMC6586289

[56] Abrahams, A., Parker, M. I. & Prince, S. The T-box transcription factor Tbx2: its role in development and possible implication in cancer. *IUBMB Life* **62**, 92–102 (2010). 19960541 10.1002/iub.275

[57] Yang, H. et al. HOXD10 acts as a tumor-suppressive factor via inhibition of the RHOC/AKT/MAPK pathway in human cholangiocellular carcinoma. *Oncol. Rep.* **34**, 1681–1691 (2015). 26260613 10.3892/or.2015.4194 PMC4564083

[58] Chang, J. W. et al. Wild-type p53 upregulates an early onset breast cancer-associated gene GAS7 to suppress metastasis via GAS7–CYFIP1-mediated signaling pathway. *Oncogene* **37**, 4137–4150 (2018). 29706651 10.1038/s41388-018-0253-9 PMC6062498

[59] Liu, S. et al. MAP2K4 interacts with Vimentin to activate the PI3K/AKT pathway and promotes breast cancer pathogenesis. *Aging (Albany NY)* **11**, 10697–10710 (2019). 31761784 10.18632/aging.102485 PMC6914392

[60] Aizawa, T. et al. Cancer-associated fibroblasts secrete Wnt2 to promote cancer progression in colorectal cancer. *Cancer Med.* **8**, 6370–6382 (2019). 31468733 10.1002/cam4.2523 PMC6797671

[61] Joshi, S. et al. Rac2 controls tumor growth, metastasis and M1–M2 macrophage differentiation in vivo. *PLoS ONE* **9**, e95893 (2014). 24770346 10.1371/journal.pone.0095893 PMC4000195

[62] Ashburner, M. et al. Gene ontology: tool for the unification of biology. The Gene Ontology Consortium. *Nat. Genet.* **25**, 25–29 (2000). 10802651 10.1038/75556 PMC3037419

[63] Gene Ontology Consortium et al. The gene ontology knowledgebase in 2023. *Genetics* **224**, iyad031 (2023). 36866529 10.1093/genetics/iyad031 PMC10158837

[64] Maddipati, R. & Stanger, B. Z. Pancreatic cancer metastases harbor evidence of polyclonality. *Cancer Discov.* **5**, 1086–1097 (2015). 26209539 10.1158/2159-8290.CD-15-0120 PMC4657730

[65] Guy, C. T., Cardiff, R. D. & Muller, W. J. Induction of mammary tumors by expression of polyomavirus middle T oncogene: a transgenic mouse model for metastatic disease. *Mol. Cell. Biol.* **12**, 954–961 (1992). 1312220 10.1128/mcb.12.3.954-961.1992 PMC369527

[66] Dekkers, J. F. et al. Long-term culture, genetic manipulation and xenotransplantation of human normal and breast cancer organoids. *Nat. Protoc.* **16**, 1936–1965 (2021). 33692550 10.1038/s41596-020-00474-1 PMC8221035

[67] Picon-Ruiz, M., Marchal, J. A. & Slingerland, J. M. Obtaining human breast adipose cells for breast cancer cell co-culture studies. *STAR Protoc.* **1**, 100197 (2020). 33377091 10.1016/j.xpro.2020.100197 PMC7757558

[68] Shalabi, S. F. et al. Evidence for accelerated aging in mammary epithelia of women carrying germline *BRCA1* or *BRCA2* mutations. *Nat. Aging* **1**, 838–849 (2021). 35187501 10.1038/s43587-021-00104-9 PMC8849557

[69] Gray, G. K. et al. A human breast atlas integrating single-cell proteomics and transcriptomics. *Dev. Cell* **57**, 1400–1420.e7 (2022). 35617956 10.1016/j.devcel.2022.05.003 PMC9202341

[70] Nyitray, C. E., Chavez, M. G. & Desai, T. A. Compliant 3D microenvironment improves β-cell cluster insulin expression through mechanosensing and β-catenin signaling. *Tissue Eng. Part A* **20**, 1888–1895 (2014). 24433489 10.1089/ten.tea.2013.0692 PMC4085995

[71] Jeong, G. S. et al. Viscoelastic lithography for fabricating self-organizing soft micro-honeycomb structures with ultra-high aspect ratios. *Nat. Commun.* **7**, 11269 (2016). 27157977 10.1038/ncomms11269 PMC4865738

[72] Girgin, M. U. et al. Bioengineered embryoids mimic post-implantation development in vitro. *Nat. Commun.* **12**, 5140 (2021). 34446708 10.1038/s41467-021-25237-8 PMC8390504

[73] Wise, K. D. & Najafi, K. Microfabrication techniques for integrated sensors and microsystems. *Science* **254**, 1335–1342 (1991). 1962192 10.1126/science.1962192

[74] Leong, T. G., Zarafshar, A. M. & Gracias, D. H. Three-dimensional fabrication at small size scales. *Small* **6**, 792–806 (2010). 20349446 10.1002/smll.200901704 PMC3078552

[75] Steedman, M. R., Tao, S. L., Klassen, H. & Desai, T. A. Enhanced differentiation of retinal progenitor cells using microfabricated topographical cues. *Biomed. Microdevices* **12**, 363–369 (2010). 20077017 10.1007/s10544-009-9392-7 PMC2859162

[76] Kharbikar, B. N., Kumar, S. H., Kr, S. & Srivastava, R. Hollow silicon microneedle array based trans-epidermal antiemetic patch for efficient management of chemotherapy induced nausea and vomiting. In *Micro + Nano Materials, Devices, and Systems* Vol. 9668 (eds Eggleton, B. J. & Palomba, S.) 256–272 (SPIE, 2015).

[77] Bernards, D. A. et al. Injectable devices for delivery of liquid or solid protein formulations. *ACS Materials Au* **3**, 255–264 (2023). 38089136 10.1021/acsmaterialsau.3c00004 PMC10176615

[78] Kharbikar, B. N., Chendke, G. S. & Desai, T. A. Modulating the foreign body response of implants for diabetes treatment. *Adv. Drug Deliv. Rev.* **174**, 87–113 (2021). 33484736 10.1016/j.addr.2021.01.011 PMC8217111

[79] Kharbikar, B. N., Mohindra, P. & Desai, T. A. Biomaterials to enhance stem cell transplantation. *Cell Stem Cell* **29**, 692–721 (2022). 35483364 10.1016/j.stem.2022.04.002 PMC10169090

[80] Shukla, L., Yuan, Y., Shayan, R., Greening, D. W. & Karnezis, T. Fat therapeutics: the clinical capacity of adipose-derived stem cells and exosomes for human disease and tissue regeneration. *Front. Pharmacol.* **11**, 158 (2020). 32194404 10.3389/fphar.2020.00158 PMC7062679

[81] White, J. D., Dewal, R. S. & Stanford, K. I. The beneficial effects of brown adipose tissue transplantation. *Mol. Aspects Med.* **68**, 74–81 (2019). 31228478 10.1016/j.mam.2019.06.004 PMC6708446

[82] Liu, X. et al. Brown adipose tissue transplantation improves whole-body energy metabolism. *Cell Res.* **23**, 851–854 (2013). 23649313 10.1038/cr.2013.64 PMC3674396

[83] Stanford, K. I. et al. Brown adipose tissue regulates glucose homeostasis and insulin sensitivity. *J. Clin. Invest.* **123**, 215–223 (2013). 23221344 10.1172/JCI62308 PMC3533266

[84] Liu, X. et al. Brown adipose tissue transplantation reverses obesity in Ob/Ob mice. *Endocrinology* **156**, 2461–2469 (2015). 25830704 10.1210/en.2014-1598

[85] Pogodzinski, D., Ostrowska, L., Smarkusz-Zarzecka, J. & Zysk, B. Secretome of adipose tissue as the key to understanding the endocrine function of adipose tissue. *Int. J. Mol. Sci.* **23**, 2309 (2022). 35216423 10.3390/ijms23042309 PMC8878787

[86] Sun, T. et al. Engineered adipose-derived stem cells overexpressing RXFP1 via CRISPR activation ameliorate erectile dysfunction in diabetic rats. *Antioxidants* **12**, 171 (2023). 36671033 10.3390/antiox12010171 PMC9854730

[87] Rudolph, M. C., Wellberg, E. A. & Anderson, S. M. Adipose-depleted mammary epithelial cells and organoids. *J. Mammary Gland Biol. Neoplasia* **14**, 381–386 (2009). 19953310 10.1007/s10911-009-9161-5 PMC4132965

[88] Currie, C. J., Poole, C. D. & Gale, E. A. The influence of glucose-lowering therapies on cancer risk in type 2 diabetes. *Diabetologia* **52**, 1766–1777 (2009). 19572116 10.1007/s00125-009-1440-6

[89] Hemkens, L. G. et al. Risk of malignancies in patients with diabetes treated with human insulin or insulin analogues: a cohort study. *Diabetologia* **52**, 1732–1744 (2009). 19565214 10.1007/s00125-009-1418-4 PMC2723679

[90] Godsland, I. F. Insulin resistance and hyperinsulinaemia in the development and progression of cancer. *Clin. Sci. (Lond)* **118**, 315–332 (2009). 19922415 10.1042/CS20090399 PMC2782313

[91] Nasiri, A. R., Rodrigues, M. R., Li, Z., Leitner, B. P. & Perry, R. J. SGLT2 inhibition slows tumor growth in mice by reversing hyperinsulinemia. *Cancer Metab.* **7**, 10 (2019). 31867105 10.1186/s40170-019-0203-1 PMC6907191

[92] Chondronikola, M. et al. Brown adipose tissue improves whole-body glucose homeostasis and insulin sensitivity in humans. *Diabetes* **63**, 4089–4099 (2014). 25056438 10.2337/db14-0746 PMC4238005

[93] Chadt, A. & Al-Hasani, H. Glucose transporters in adipose tissue, liver, and skeletal muscle in metabolic health and disease. *Pflugers Arch.* **472**, 1273–1298 (2020). 32591906 10.1007/s00424-020-02417-x PMC7462924

[94] Kousteni, S. FoxO1, the transcriptional chief of staff of energy metabolism. *Bone* **50**, 437–443 (2012). 21816244 10.1016/j.bone.2011.06.034 PMC3228887

[95] Schilperoort, M. et al. The GPR120 agonist TUG-891 promotes metabolic health by stimulating mitochondrial respiration in brown fat. *EMBO Mol. Med.* **10**, e8047 (2018). 29343498 10.15252/emmm.201708047 PMC5840546

[96] Satapati, S. et al. GPR120 suppresses adipose tissue lipolysis and synergizes with GPR40 in antidiabetic efficacy. *J. Lipid Res.* **58**, 1561–1578 (2017). 28583918 10.1194/jlr.M075044 PMC5538279

[97] Nguyen, H. P. et al. Aifm2, a NADH oxidase, supports robust glycolysis and is required for cold- and diet-induced thermogenesis. *Mol. Cell* **77**, 600–617.e4 (2020). 31952989 10.1016/j.molcel.2019.12.002 PMC7031813

[98] Wang, P., Mariman, E., Renes, J. & Keijer, J. The secretory function of adipocytes in the physiology of white adipose tissue. *J. Cell. Physiol.* **216**, 3–13 (2008). 18264975 10.1002/jcp.21386

[99] Bond, S. T., Calkin, A. C. & Drew, B. G. Adipose-derived extracellular vesicles: systemic messengers and metabolic regulators in health and disease. *Front. Physiol.* **13**, 837001 (2022). 35283789 10.3389/fphys.2022.837001 PMC8905439

[100] Matharu, N. et al. CRISPR-mediated activation of a promoter or enhancer rescues obesity caused by haploinsufficiency. *Science* **363**, eaau0629 (2019). 30545847 10.1126/science.aau0629 PMC6570489

[101] Wang, X. et al. Evaluation and optimization of differentiation conditions for human primary brown adipocytes. *Sci. Rep.* **8**, 5304 (2018). 29593245 10.1038/s41598-018-23700-z PMC5871774

[102] Lowell, B. B. & Flier, J. S. Brown adipose tissue, β3-adrenergic receptors, and obesity. *Annu. Rev. Med.* **48**, 307–316 (1997). 9046964 10.1146/annurev.med.48.1.307

[103] Sun, X. et al. Mirabegron displays anticancer effects by globally browning adipose tissues. *Nat. Commun.* **14**, 7610 (2023). 37993438 10.1038/s41467-023-43350-8 PMC10665320

[104] Chen, J., Guo, Z., Tian, H. & Chen, X. Production and clinical development of nanoparticles for gene delivery. *Mol. Ther. Methods Clin. Dev.* **3**, 16023 (2016). 27088105 10.1038/mtm.2016.23 PMC4822651

[105] Banskota, S. et al. Engineered virus-like particles for efficient in vivo delivery of therapeutic proteins. *Cell* **185**, 250–265.e16 (2022). 35021064 10.1016/j.cell.2021.12.021 PMC8809250

[106] Tsagkaraki, E. et al. CRISPR-enhanced human adipocyte browning as cell therapy for metabolic disease. *Nat. Commun.* **12**, 6931 (2021). 34836963 10.1038/s41467-021-27190-y PMC8626495

[107] Steele, C. B. et al. Vital signs: trends in incidence of cancers associated with overweight and obesity—United States, 2005–2014. *MMWR Morb. Mortal Wkly Rep.* **66**, 1052–1058 (2017). 28981482 10.15585/mmwr.mm6639e1 PMC5720881

[108] Renehan, A. G., Zwahlen, M. & Egger, M. Adiposity and cancer risk: new mechanistic insights from epidemiology. *Nat. Rev. Cancer* **15**, 484–498 (2015). 26205341 10.1038/nrc3967

[109] Paz-Filho, G., Lim, E. L., Wong, M. L. & Licinio, J. Associations between adipokines and obesity-related cancer. *Front. Biosci. (Landmark Ed.)* **16**, 1634–1650 (2011). 21196253 10.2741/3810

[110] Park, J., Morley, T. S., Kim, M., Clegg, D. J. & Scherer, P. E. Obesity and cancer—mechanisms underlying tumour progression and recurrence. *Nat. Rev. Endocrinol.* **10**, 455–465 (2014). 24935119 10.1038/nrendo.2014.94 PMC4374431

[111] Sirin, O. & Kolonin, M. G. Treatment of obesity as a potential complementary approach to cancer therapy. *Drug Discov. Today* **18**, 567–573 (2013). 22627005 10.1016/j.drudis.2012.05.008

[112] Khandekar, M. J., Cohen, P. & Spiegelman, B. M. Molecular mechanisms of cancer development in obesity. *Nat. Rev. Cancer* **11**, 886–895 (2011). 22113164 10.1038/nrc3174

[113] Huffman, D. M. et al. Cancer progression in the transgenic adenocarcinoma of mouse prostate mouse is related to energy balance, body mass, and body composition, but not food intake. *Cancer Res.* **67**, 417–424 (2007). 17185379 10.1158/0008-5472.CAN-06-1244

[114] Zhang, Y. et al. Stromal progenitor cells from endogenous adipose tissue contribute to pericytes and adipocytes that populate the tumor microenvironment. *Cancer Res.* **72**, 5198–5208 (2012). 23071132 10.1158/0008-5472.CAN-12-0294

[115] Wang, Y. Y. et al. Mammary adipocytes stimulate breast cancer invasion through metabolic remodeling of tumor cells. *JCI Insight* **2**, e87489 (2017). 28239646 10.1172/jci.insight.87489 PMC5313068

[116] Rohrig, F. & Schulze, A. The multifaceted roles of fatty acid synthesis in cancer. *Nat. Rev. Cancer* **16**, 732–749 (2016). 27658529 10.1038/nrc.2016.89

[117] Beloribi-Djefaflia, S., Vasseur, S. & Guillaumond, F. Lipid metabolic reprogramming in cancer cells. *Oncogenesis* **5**, e189 (2016). 26807644 10.1038/oncsis.2015.49 PMC4728678

[118] Ye, H. et al. Leukemic stem cells evade chemotherapy by metabolic adaptation to an adipose tissue niche. *Cell Stem Cell* **19**, 23–37 (2016). 27374788 10.1016/j.stem.2016.06.001 PMC4938766

[119] Cypess, A. M. Reassessing human adipose tissue. *N. Engl. J. Med.* **386**, 768–779 (2022). 35196429 10.1056/NEJMra2032804

[120] Bashor, C. J., Hilton, I. B., Bandukwala, H., Smith, D. M. & Veiseh, O. Engineering the next generation of cell-based therapeutics. *Nat. Rev. Drug Discov.* **21**, 655–675 (2022). 35637318 10.1038/s41573-022-00476-6 PMC9149674

[121] Chendke, G. S. et al. Replenishable prevascularized cell encapsulation devices increase graft survival and function in the subcutaneous space. *Bioeng. Transl. Med.* **8**, e10520 (2023). 37476069 10.1002/btm2.10520 PMC10354771

[122] Kharbikar, B. N., Zhong, J. X., Cuylear, D. L., Perez, C. A. & Desai, T. A. Theranostic biomaterials for tissue engineering. *Curr. Opin. Biomed. Eng.* **19**, 100299 (2021). 35529078 10.1016/j.cobme.2021.100299 PMC9075690

[123] Herberts, C. A., Kwa, M. S. & Hermsen, H. P. Risk factors in the development of stem cell therapy. *J. Transl. Med.* **9**, 29 (2011). 21418664 10.1186/1479-5876-9-29 PMC3070641

[124] Buitinga, M. et al. Micro-fabricated scaffolds lead to efficient remission of diabetes in mice. *Biomaterials* **135**, 10–22 (2017). 28478326 10.1016/j.biomaterials.2017.03.031

[125] Carpenter, R. et al. Scaffold-assisted ectopic transplantation of internal organs and patient-derived tumors. *ACS Biomater. Sci. Eng.* **5**, 6667–6678 (2019). 33423485 10.1021/acsbiomaterials.9b00978 PMC7808342

[126] Cordeiro, P. G. Breast reconstruction after surgery for breast cancer. *N. Engl. J. Med.* **359**, 1590–1601 (2008). 18843123 10.1056/NEJMct0802899

[127] Juntunen, M. et al. Evaluation of the effect of donor weight on adipose stromal/stem cell characteristics by using weight-discordant monozygotic twin pairs. *Stem Cell Res. Ther.* **12**, 516 (2021). 34565451 10.1186/s13287-021-02587-0 PMC8474937

[128] Chu, D. T. et al. Adipose tissue stem cells for therapy: an update on the progress of isolation, culture, storage, and clinical application. *J. Clin. Med.* **8**, 917 (2019). 31247996 10.3390/jcm8070917 PMC6678927

[129] Doench, J. G. et al. Optimized sgRNA design to maximize activity and minimize off-target effects of CRISPR–Cas9. *Nat. Biotechnol.* **34**, 184–191 (2016). 26780180 10.1038/nbt.3437 PMC4744125

[130] Schindelin, J. et al. Fiji: an open-source platform for biological-image analysis. *Nat. Methods* **9**, 676–682 (2012). 22743772 10.1038/nmeth.2019 PMC3855844

[131] Dobin, A. et al. STAR: ultrafast universal RNA-seq aligner. *Bioinformatics* **29**, 15–21 (2013). 23104886 10.1093/bioinformatics/bts635 PMC3530905

[132] Law, C. W., Chen, Y., Shi, W. & Smyth, G. K. voom: precision weights unlock linear model analysis tools for RNA-seq read counts. *Genome Biol.* **15**, R29 (2014). 24485249 10.1186/gb-2014-15-2-r29 PMC4053721

[133] H. P. Nguyen, et al. Project ID GSE246231. *Gene Expression Omnibus* https://www.ncbi.nlm.nih.gov/geo/query/acc.cgi?acc=GSE246231 (2024).

[134] Qiu, W. & Su, G. H. Development of orthotopic pancreatic tumor mouse models. *Methods Mol. Biol.* **980**, 215–223 (2013). 23359156 10.1007/978-1-62703-287-2_11 PMC4049460

[135] Rosenbluth, J. M. et al. Organoid cultures from normal and cancer-prone human breast tissues preserve complex epithelial lineages. *Nat. Commun.* **11**, 1711 (2020). 32249764 10.1038/s41467-020-15548-7 PMC7136203

