# Supplementary material for: Implantation of engineered adipocytes suppresses tumor progression in cancer models
Source: Nat Biotechnol. 2025 Feb 4;43(12):1979–95. doi: 10.1038/s41587-024-02551-2 (PMC12319119; doi:10.1038/s41587-024-02551-2)
Supplement: Supplementary file 1 — Supplementary Tables 2–4. [file 41587_2024_2551_MOESM1_ESM.pdf]

nature biotechnology

Supplementary information https://doi.org/10.1038/s41587-024-02551-2

Implantation of engineered adipocytes suppresses tumor progression in cancer models

In the format provided by the authors and unedited

Supplementary Table 2. Breast cancer organoid characteristics.

**Name**	**ER**	**PR**	**HER2**	**Metastatic**	**Inflammatory?**	**Source of adipocytes?**
**TOR40**	Negative	Negative	Negative	no	yes	ad-1 (unmatched breast)
**TOR120**	Negative	Negative	Negative	yes	no	ad-2 (unmatched breast)
**TOR127**	Positive	Positive	Negative	yes	no	ad-3 (unmatched breast)
**TOR124**	Negative	Negative	Negative	yes	no	ad-4 (unmatched breast)
**TOR41**	Negative	Negative	Negative	no	yes	ad-5 (matched breast)

**Name**	**Gene**	**Germline mutation**	**Cancer history**	**Source of adipocytes?**
ORG150	*BRCA2*	6174delT	Two breast cancers	ad-6 (matched breast)
ORG158	*BRCA1*	c.5266dup	Ductal carcinoma in situ (DCIS)	ad-7 (matched breast)
ORG164	*RAD51D*	c.270_271dupTA	Breast cancer	ad-8 (matched breast)

Supplementary Table 3. Sequences of primers for RT-qPCR, cloning, genotyping and gRNAs.

Gene/Use/sgRNA	Primers
GAPDH	GTC TCC TCT GAC TTC AAC AGC G
	ACC ACC CTG TTG CTG TAG CCA A
UCP1	TGG AAT AGC GGC GTG CTT G
	CTC ATC AGA TTG GGA GTA G
PPARGC1A	CCA AAG GAT GCG CTC TCG TTC A
	CGG TGT CTG TAG TGG CTT GAC T
ADIPOQ	CAG GCC GTG ATG GCA GAG ATG
	GGT TTC ACC GAT GTC TCC CTT AG
PLIN1	GCG GAA TTT GCT GCC AAC ACT C
	AGA CTT CTG GGC TTG CTG GTG T
FABP4	ACG AGA GGA TGA TAA ACT GGT GG
	GCG AAC TTC AGT CCA GGT CAA C
MKI67	GAA AGA GTG GCA ACC TGC CTT C
	GCA CCA AGT TTT ACT ACA TCT GCC
CD36	CAG GTC AAC CTA TTG GTC AAG CC
	GCC TTC TCA TCA CCA ATG GTC C
GLUT4	TGG GCT TCT TCA TCT TCA CC
	GTG CTG GGT TTC ACC TCC T
GCK	CAT CTC CGA CTT CCT GGA CAA G
	TGG TCC AGT TGA GAA GGA TGC C
CPT1b	TGT ATC GCC GTA AAC TGG ACC G
	TGT CTG AGA GGT GCT GTA GCA C
TFAM	GTG GTT TTC ATC TGT CTT GGC AAG
	TTC CCT CCA ACG CTG GGC AAT T
DIO2	TTG AGC CGC TCC AAG TCC ACT C
	CTG TAC TGG AGA CAT GCA CCA C
Gapdh	TGC ACC ACC AAC TGC TTA G
	GGA TGC AGG GAT GAT GTT C
Ucp1	GTG AAC CCG ACA ACT TCC GAA
	TGA AAC TCC GGC TGA GAA GAT
Ppargc1a	ACA GCT TTC TGG GTG GAT TG
	TGT CTC TGT GAG GAC CGC TA
Prdm16	TAT GGA GTG ACA TAG AGT GTG CT
	CCA CTT CAA TCC ACC CAG AAA G
Glut4	GTA ACT TCA TTG TCG GCA TGG
	AGC TGA GAT CTG GTC AAA CG
Gck	GCA TCT CTG ACT TCC TGG ACA AG
	CTT GGT CCA GTT GAG CAG GAT G
Cd36	GGA CAT TGA GAT TCT TTT CCT CTG
	GCA AAG GCA TTG GCT GGA AGA AC
Cpt1b	ATG TAT CGC CGC AAA CTG GAC C

	CTC TGA GAG GTG CTG TAG CAA G
Mki67	GAG GAG AAA CGC CAA CCA AGA G
	TTT GTC CTC GGT GGC GTT ATC C
MTOR	
	AGC ATC GGA TGC TTA GGA GTG G
	CAG CCA GTC ATC TTT GGA GAC C
CK5	ACG TCC ACT CTG CGA GAA TG<br>CGA TCA GGC TGT ACG TCA TCT
CK8	ACA AGG TAG AGC TGG AGT CTC G
	AGC ACC ACA GAT GTG TCC GAG A
CK17	ATC CTG CTG GAT GTG AAG ACG C
	TCC ACA ATG GTA CGC ACC TGA C
UPP1	CTC CAA CGT CAC TAT CAT CCG C
	CTG CCT TGA AGC AGG TAT CCA C
Cas9	ATC ACCC CCC ACC AGA TCA AGC
	GTC CTT GTC GTA CAG GCC GTT CA
UCP1 gRNA-1	CCCGAGGCACCGAGCGAGAAT
UCP1 gRNA-2	GCAGGGCTCCCGAGGCACCGA
UCP1 gRNA-3	AGGGCTCCCGAGGCACCGAGC
UCP1 gRNA-4	ACCGAGCGAGAATGGGAATGG
UCP1 gRNA-5	GCACCGAGCGAGAATGGGAAT
PPARGC1a gRNA-1	CACTGAAGCAGAGGGCTGCCT
PPARGC1a gRNA-2	TTAGAGCAGCAAGCTGCACAG
PPARGC1a gRNA-3	GTTAGAGCAGCAAGCTGCACA
PPARGC1a gRNA-4	GGGCTGCCTTTGAGTGACGTC
PPARGC1a gRNA-5	AGTTAGAGCAGCAAGCTGCAC
PRDM16 gRNA-1	CGGCGGCGGCGGCGCGACGAT
PRDM16 gRNA-2	GGCGGCGCGACGATGAGGATG
PRDM16 gRNA-3	GCCGCCGCCGCCGCCTCGGCG
PRDM16 gRNA-4	CGCCGCCGCCGCCGCCTCGGC
PRDM16 gRNA-5	GGCGGCGGCGGCGGCGCGACG
Ucp1 gRNA-1	GGGACTTGGCAGGGGCGTGCC
Ucp1 gRNA-2	CTGAGCCGGCCCAGGTCTCCA
Ucp1 gRNA-3	CAGGTCTCCAAAGAGCTGCTA
Ucp1 gRNA-4	CTTTGGGAGTGACGCGCGGCT
Ucp1 gRNA-5	GGCTTTGGGAGTGACGCGCGG
UPP1 gRNA-1	GCGAACTCAGCTGACCGCATC
UPP1 gRNA-2	GGGCTTGTCTGCGGGATGGCC
UPP1 gRNA-3	GATGGCCCAGGATGCGGTCAG
UPP1 gRNA-4	GGTCAGCTGAGTTCGCCGGCC
UPP1 gRNA-5	AGTTCGCCGGCCCAGGGCAGG

Supplementary Table 4. List of antibodies.

Name	Vendor	Concentration	Catalog number
Ki67 (SolA15)	Fisher Scientific	5ug/ml	14-5698-82
Carbonic Anhydrase	Fisher Scientific	3ug/ml	AF2188
CD31	Fisher Scientific	10ug/ml	BBA7
Caspase 3	Cell Signaling	1:400	9661
CK19	Abcam	1:200	Ab203444
GAPDH	Cell Signaling	1:1000	5174
UCP1	R&D	0.5ug/ml	MAB6158
Goat anti-rat, Alexa Flour 647	Fisher Scientific	1:1000	A21247
Goat anti-mouse, Alexa Fluor 594	Life Technologies	1:500	A11032
Donkey anti-goat, Alexa Fluor 594	Fisher Scientific	1:1000	A11055
