# Supplementary material for: Implantation of engineered adipocytes suppresses tumor progression in cancer models
Source: Nat Biotechnol. 2025 Feb 4;43(12):1979–95. doi: 10.1038/s41587-024-02551-2 (PMC12319119; doi:10.1038/s41587-024-02551-2)
Supplement: Supplementary file 2 — Reporting Summary [file 41587_2024_2551_MOESM2_ESM.pdf]

nature portfolio

Corresponding author(s): Nadav Ahituv

Last updated by author(s): 12/14/2024

Reporting Summary

Nature Portfolio wishes to improve the reproducibility of the work that we publish. This form provides structure for consistency and transparency in reporting. For further information on Nature Portfolio policies, see our Editorial Policies and the Editorial Policy Checklist.

Statistics

For all statistical analyses, confirm that the following items are present in the figure legend, table legend, main text, or Methods section.

n/a	Confirmed	
☐	☒	The exact sample size (*n*) for each experimental group/condition, given as a discrete number and unit of measurement
☐	☒	A statement on whether measurements were taken from distinct samples or whether the same sample was measured repeatedly
☐	☒	The statistical test(s) used AND whether they are one- or two-sided<br>*Only common tests should be described solely by name; describe more complex techniques in the Methods section.*
☒	☐	A description of all covariates tested
☒	☐	A description of any assumptions or corrections, such as tests of normality and adjustment for multiple comparisons
☐	☒	A full description of the statistical parameters including central tendency (e.g. means) or other basic estimates (e.g. regression coefficient) AND variation (e.g. standard deviation) or associated estimates of uncertainty (e.g. confidence intervals)
☐	☒	For null hypothesis testing, the test statistic (e.g. *F*, *t*, *r*) with confidence intervals, effect sizes, degrees of freedom and *P* value noted<br>*Give P values as exact values whenever suitable.*
☒	☐	For Bayesian analysis, information on the choice of priors and Markov chain Monte Carlo settings
☒	☐	For hierarchical and complex designs, identification of the appropriate level for tests and full reporting of outcomes
☒	☐	Estimates of effect sizes (e.g. Cohen's *d*, Pearson's *r*), indicating how they were calculated

Our web collection on statistics for biologists contains articles on many of the points above.

Software and code

Policy information about availability of computer code

Data collection	N.A
Data analysis	Partek Flow

For manuscripts utilizing custom algorithms or software that are central to the research but not yet described in published literature, software must be made available to editors and reviewers. We strongly encourage code deposition in a community repository (e.g. GitHub). See the Nature Portfolio guidelines for submitting code & software for further information.

Data

Policy information about availability of data

All manuscripts must include a data availability statement. This statement should provide the following information, where applicable:

- Accession codes, unique identifiers, or web links for publicly available datasets
- A description of any restrictions on data availability
- For clinical datasets or third party data, please ensure that the statement adheres to our policy

RNA-seq is available as GEO accession number GSE246231.

Research involving human participants, their data, or biological material

Policy information about studies with human participants or human data. See also policy information about sex, gender (identity/presentation), and sexual orientation and race, ethnicity and racism.

Reporting on sex and gender	N.A
Reporting on race, ethnicity, or other socially relevant groupings	N.A
Population characteristics	N.A
Recruitment	N.A
Ethics oversight	N.A

Note that full information on the approval of the study protocol must also be provided in the manuscript.

Field-specific reporting

Please select the one below that is the best fit for your research. If you are not sure, read the appropriate sections before making your selection.

☒ Life sciences ☐ Behavioural & social sciences ☐ Ecological, evolutionary & environmental sciences

For a reference copy of the document with all sections, see nature.com/documents/nr-reporting-summary-flat.pdf

Life sciences study design

All studies must disclose on these points even when the disclosure is negative.

Sample size	Sample size of at least 2 for cells or 4 for mice was selected based on variance observed in prior experiments of a similar nature, as well as practical considerations.
Data exclusions	Data were not exclude from analysis.
Replication	To ensure robust reproducibility, all data presented in the manuscript were repeated at least three times (two times only for initial CRISPRa sgRNA transfection guide selection). All replication attempts were successful.
Randomization	N.A
Blinding	No blinding was performed in this study. It required to use the correct genotypes of cells and mice

Reporting for specific materials, systems and methods

We require information from authors about some types of materials, experimental systems and methods used in many studies. Here, indicate whether each material, system or method listed is relevant to your study. If you are not sure if a list item applies to your research, read the appropriate section before selecting a response.

Materials & experimental systems

n/a	Involved in the study	
☐	☒	Antibodies
☐	☒	Eukaryotic cell lines
☒	☐	Palaeontology and archaeology
☐	☒	Animals and other organisms
☒	☐	Clinical data
☒	☐	Dual use research of concern
☒	☐	Plants

Methods

n/a	Involved in the study	
☒	☐	ChIP-seq
☒	☐	Flow cytometry
☒	☐	MRI-based neuroimaging

Antibodies

Antibodies used	Ki67 (SolA15),Fisher Scientific,14-5698-82<br>Carbonic Anhydrase,Fisher Scientific,AF2188<br>CD31,Fisher Scientific,BBA7

	GAPDH, Cell Signaling, 5174<br>Goat anti-rat, Alexa Flour 647,Fisher Scientific,A21247<br>Goat anti-mouse, Alexa Fluor 594,Life Technologies,A11032<br>Donkey anti-goat, Alexa Fluor 594,Fisher Scientific,A11055<br>Caspase 3, Cell Signaling, CS9661<br>CK19, Abcam, Ab203444<br>UCP1, R&D, MAB6158
Validation	Ki67- https://www.thermofisher.com/antibody/product/Ki-67-Antibody-clone-SolA15-Monoclonal/14-5698-37?gclid=Cj0KCQjw27mhBhC9ARIsAIFsETEvjm59r_6344DC3_jxeeeIBcjHtvUN6FV_e1JxDJyqy8Z0Ns9dsaIaAhu-EALw_wcB&ef_id=Cj0KCQjw27mhBhC9ARIsAIFsETEvjm59r_6344DC3_jxeeeIBcjHtvUN6FV_e1JxDJyqy8Z0Ns9dsaIaAhu-EALw_wcB:G:s&s_kwcid=AL!3652!3!278870232429!!!g!!!1454324556!63404918784&cid=bid_pca_frg_r01_co_cp1359_pjt0000_bid00000_0se_gaw_dy_pur_con.<br>CA9-https://www.rndsystems.com/products/human-carbonic-anhydrase-ix-ca9-antibody_af2188<br>CD31-https://www.rndsystems.com/products/human-cd31-pecam-1-antibody-9g11_bba7?gclid=Cj0KCQjw27mhBhC9ARIsAIFsETESGafo_oT07Q0oQgcgGzrDCU8jmY4a2yrvbTsHU1rGsCo_-SFX41YaAsWdEALw_wcB&gclsrc=aw.ds<br>Caspase3-https://www.cellsignal.com/products/primary-antibodies/cleaved-caspase-3-asp175-antibody/9661<br>CK19- https://www.abcam.com/products/primary-antibodies/alexa-fluor-555-cytokeratin-19-antibody-ep1580y-ab203444.html<br>GAPDH- https://www.cellsignal.com/products/primary-antibodies/gapdh-d16h11-xp-rabbit-mab/5174<br>UCP1- https://www.rndsystems.com/products/human-mouse-ucp1-antibody-536435_mab6158

Eukaryotic cell lines

Policy information about cell lines and Sex and Gender in Research

Cell line source(s)	Human preadipocyte cell line was obtained from Dr. Hei Sook Sul lab at UC Berkeley. It was generated by immortalizing human male subcutaneous preadipocytes. DU-415, Panc 10.01, SW-1417, MDA-MB-436, MCF7 and Mouse 3T3-L1 were obtained from ATCC. AAVpro 293T cells were obtained from Takara.
Authentication	Preadipocyte cell lines were subjected adipocyte differentiation and adipogenic markers were checked with qRT-PCR.
Mycoplasma contamination	All lines were tested for mycoplasma.
Commonly misidentified lines<br>(See ICLAC register)	N.A

Animals and other research organisms

Policy information about studies involving animals; ARRIVE guidelines recommended for reporting animal research, and Sex and Gender in Research

Laboratory animals	SCID mice (JAX, 001303), FVB/N-Tg(MMTV-PyVT)634Mul/J (JAX, 002374), Krastm4Tyj Trp53tm1Brn Tg(Pdx1-cre/Esr1*)#Dam/J (Jax, 032429)
Wild animals	N.A
Reporting on sex	We used both males and females
Field-collected samples	N.A
Ethics oversight	UCSF Institutional Animal Care & Use Program

Note that full information on the approval of the study protocol must also be provided in the manuscript.
