# Supplementary material for: Implantation of engineered adipocytes suppresses tumor progression in cancer models
Source: Nat Biotechnol. 2025 Feb 4;43(12):1979–95. doi: 10.1038/s41587-024-02551-2 (PMC12319119; doi:10.1038/s41587-024-02551-2)
Supplement: Supplementary file 4 — Source Data Fig. 1. Raw data for western blots. a,b, Uncropped images of the western blot displayed in Extended Data Fig. 1l. The same blot was cut and stained on the left for GAPDH (a) and UCP1 on the right (b) because both proteins were close in size (36 kDa for GAPDH and 33 kDa for UCP1). GAPDH was used as a loading marker because of its use also in RT–qPCR. A total of 10 µg per lane GAPDH, 40 µg per lane dCas9–VP64 only (VP64 (neg.)), 40 µg per lane dCas9–VP64 + UCP1-sgRNA (UCP1-CRISPRa) and 10 µg per lane UCP1 overexpression were loaded in their respective lanes. c, Additional western blot using the same samples in a and b showing 40 µg per lane dCas9–VP64 only (VP64 (neg.)), 40 µg per lane dCas9–VP64 + UCP1-sgRNA (UCP1-CRISPRa) and 10 µg per lane UCP1 overexpression. The PageRuler Plus Protein Ladder (Thermo Fisher) was used as a marker in all western blots. [file 41587_2024_2551_MOESM4_ESM.pdf]

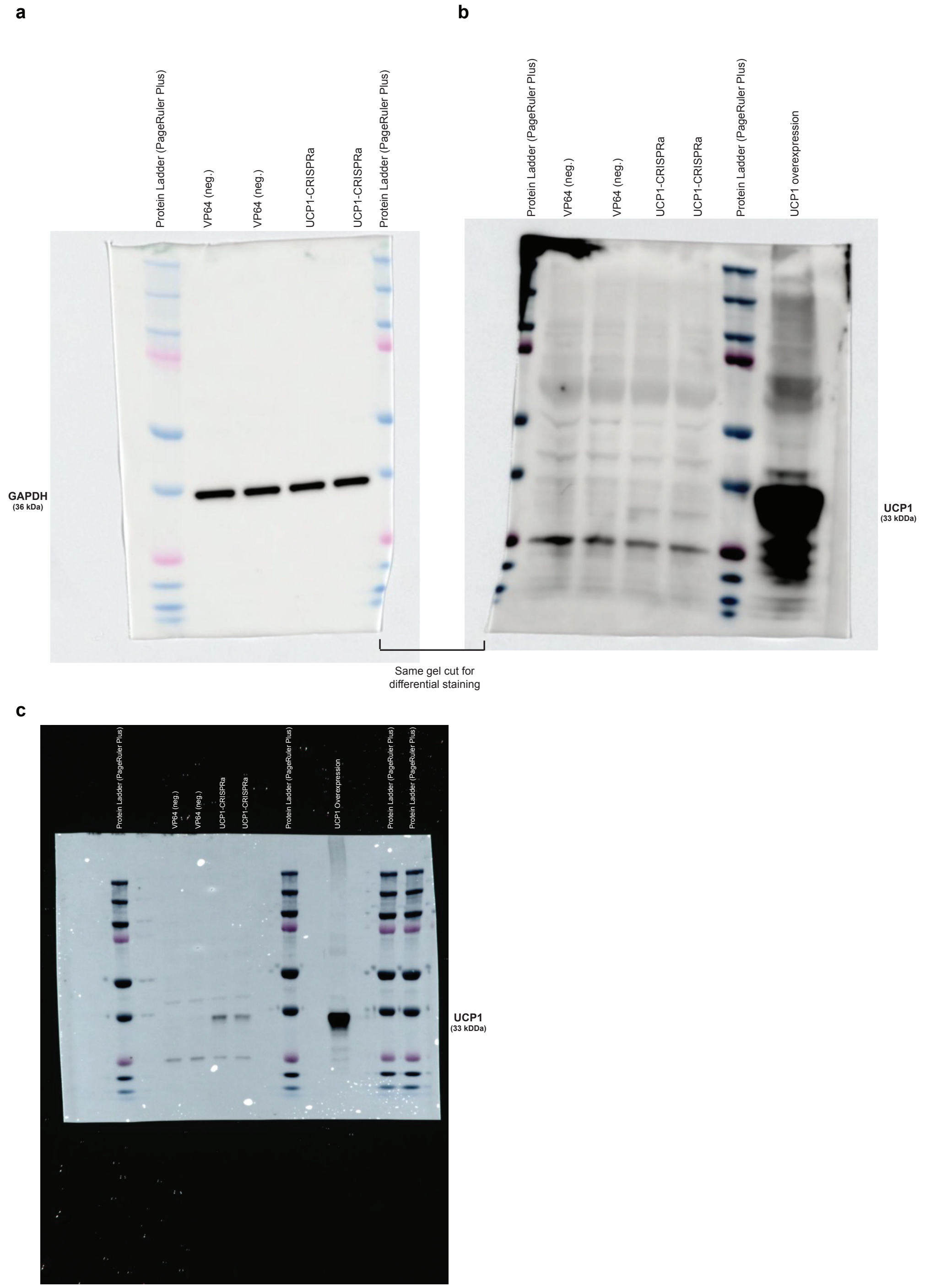

Source Data Figure 1. Raw data for Western blots. a-b, Uncropped images of the Western blot displayed in **Extended Data Figure 1l**. The same blot was cut and stained on the left for GAPDH (**a**) and UCP1 on the right (**b**) due to both proteins being close in size (36 kDa for GAPDH and 33 kDa for UCP1). GAPDH was used as a loading marker due to its use also in qRT-PCR. 10ug/lane GAPDH, 40ug/lane dCas9-VP64 only (VP64 (neg.)), 40ug/lane dCas9-VP64 + UCP1-sgRNA (UCP1-CRISPRa) and 10ug/lane UCP1 overexpression were loaded in their respective lanes. **c**, Additional Western blot using the same samples in a-b showing 40ug/lane dCas9-VP64 only (VP64 (neg.)), 40ug/lane dCas9-VP64 + UCP1-sgRNA (UCP1-CRISPRa) and 10ug/lane UCP1 overexpression. The PageRuler Plus Protein Ladder (Thermo Fisher) was used as a marker in all Westerns.
